# A real-time spatio-temporal syndromic surveillance system with application to small companion animals

**DOI:** 10.1038/s41598-019-53352-6

**Published:** 2019-11-28

**Authors:** Alison C. Hale, Fernando Sánchez-Vizcaíno, Barry Rowlingson, Alan D. Radford, Emanuele Giorgi, Sarah J. O’Brien, Peter J. Diggle

**Affiliations:** 1 Centre for Health Informatics, Computing, and Statistics (CHICAS), Lancaster Medical School, Lancaster University, Lancaster, LA1 4YW UK; 2 Bristol Veterinary School, Langford Campus, University of Bristol, Bristol, BS40 5DU UK; 3 NIHR Health Protection Research Unit in Emerging and Zoonotic Infections, University of Liverpool, Liverpool, UK; 4 Department of Infection Biology, Institute of Infection and Global Health, Leahurst Campus, University of Liverpool, Neston, CH64 7TE UK; 5 Department of Public Health and Policy, Institute of Psychology Health and Society, The Farr Institute@HeRC, University of Liverpool, Liverpool, L69 3GL UK; 6 NIHR Health Protection Research Unit in Gastrointestinal Infections, University of Liverpool, Liverpool, UK

**Keywords:** Disease prevention, Epidemiology, Digestive signs and symptoms, Scientific data, Statistics

## Abstract

Lack of disease surveillance in small companion animals worldwide has contributed to a deficit in our ability to detect and respond to outbreaks. In this paper we describe the first real-time syndromic surveillance system that conducts integrated spatio-temporal analysis of data from a national network of veterinary premises for the early detection of disease outbreaks in small animals. We illustrate the system’s performance using data relating to gastrointestinal disease in dogs and cats. The data consist of approximately one million electronic health records for dogs and cats, collected from 458 UK veterinary premises between March 2014 and 2016. For this illustration, the system predicts the relative reporting rate of gastrointestinal disease amongst all presentations, and updates its predictions as new data accrue. The system was able to detect simulated outbreaks of varying spatial geometry, extent and severity. The system is flexible: it generates outcomes that are easily interpretable; the user can set their own outbreak detection thresholds. The system provides the foundation for prompt detection and control of health threats in companion animals.

## Introduction

Surveillance systems have been developed globally for animal and/or public health purposes, facilitating the prevention and control of disease or infection nationally and regionally. During the past decade, the emergence of new diseases^1^ and the increasing threat of bio-terrorism have motivated the development of syndromic surveillance systems in public health focused on the early detection of health threats that require effective public health action^2,3^. Syndromic surveillance uses health-related data that precedes diagnosis. Although data of this kind are less specific than data from confirmed diagnoses they are typically more timely, which is an important consideration for real-time or near-real-time analysis and interpretation^4^. In veterinary medicine the development of systems for early health-event detection has followed a similar path to that previously taken in public health^5^. A recent inventory of current and planned European veterinary syndromic surveillance systems showed wide interest in European countries for syndromic surveillance, but also highlighted the novelty of this field^6^.

Small companion animal populations largely lack co-ordinated national and international disease surveillance. This has produced a deficit in our understanding of the dynamics and burden of the full range of endemic/emerging diseases in companion animals and leaves these populations susceptible to the emergence of health threats. Lack of disease surveillance also has implications for human health, as approximately 75 percent of new and emerging diseases are zoonotic^7^. However, as health records become digitised in veterinary practices they become more available for research^8^, providing an opportunity to improve companion animal syndromic surveillance in clinical settings and the possibility of linking this with human syndromic surveillance. Recently, electronic syndromic surveillance data on companion animals has become available in real-time on a national scale in the UK through surveillance schemes such as the Small Animal Veterinary Surveillance Network (SAVSNET)^9^. SAVSNET harnesses the growing volume of patient electronic health records (EHRs) available from small animal practices and complementary data from diagnostic laboratories to improve animal and human health through rapid and actionable research and surveillance.

Here we propose a real-time syndromic surveillance system that uses a spatio-temporal model in conjunction with Bayesian inference for the early detection of health-event outbreaks. Specifically, we use a Markov Chain Monte Carlo (MCMC) algorithm to generate samples from the Bayesian predictive distribution of the underlying spatio-temporal surface. These samples are then used to compute predictive probabilities at given thresholds; a high predictive probability at a particular location and time gives an early warning of a possible disease outbreak. The system provides end-users (i.e. practising veterinary surgeons) decision-support tools for immediate analysis and easy interpretation of their data. As an example, we apply our model to small companion animal EHRs collected over two years by SAVSNET from a large network of UK veterinary premises. We illustrate the feasibility of our proposed surveillance system using gastrointestinal (GI) disease in dogs and cats as an example.

Gastrointestinal (GI) disease is one of the four syndromes for which SAVSNET currently gathers information for every consultation it receives. GI disease affects animal welfare, can be expensive to manage and may be transmissible to other pets^10^ or, more rarely, to people^11^. Current approaches to preventing and controlling GI disease in companion animals have focussed on individuals or small groups of animals. This seems to have had little impact on GI disease, which remains one of the commonest reasons for presenting for veterinary care in the UK^9,10,12–15^, although precise data to confirm this has been lacking. A more coordinated population-scale approach to GI disease surveillance in companion animals is needed.

This paper focuses on the early detection of a GI disease *outbreak*, which we define as an unexplained, spatially and temporally localised increase in the fraction of GI consultations amongst all consultations. We illustrate the performance of our proposed surveillance system on simulated GI disease outbreaks of varying spatial extent and severity. This is, to our knowledge, the first surveillance system that conducts integrated spatio-temporal analysis of data from a national network of veterinary practices so as to enable real-time detection of spatially and temporally localised changes in reporting patterns across the network.

The paper is structured as follows. First, we give details of the SAVSNET and socioeconomic data used in this paper. We then give the rationale for our methodological approach, describe the spatio-temporal stochastic model that is the foundation of our surveillance system, and report the results of fitting our model to our SAVSNET-acquired data. We then simulate spatio-temporal GI outbreaks by perturbing the actual SAVSNET data in various ways to demonstrate the ability of the surveillance system to achieve timely outbreak-detection. Finally, we discuss the similarities and differences between our proposed system and other approaches in the literature, and also extensions for joint human and veterinary surveillance.

## Data Sources

### Savsnet

#### Data collection

Data were collected electronically in near-real-time from volunteer veterinary premises or sites using a compatible version of the practice management system (PMS) namely RoboVet (VetSolutions, Edinburgh) and Teleos Systems Ltd (Birmingham). This study used data for dogs and cats collected over the period between 1^st^ March 2014 and 29^th^ February 2016. In our analysis we included data from an increasing number of premises as they enrolled in the RoboVet and Teleos systems. By 29^th^ February 2016 we had data from 216 practices (amounting to a total of 458 distinct premises) located in England, Wales and Scotland. The data were extracted from consultations where a booked appointment was made to see a veterinary surgeon or nurse, including out-of-hours consultations. Through the SAVSNET system a compulsory, single-question questionnaire is appended at the end of each consultation allowing the attending veterinary surgeon or nurse to categorise the main reason for the animal’s presentation into syndromes (currently GI disease, respiratory disease, pruritus and renal disease) or other routine veterinary interventions (i.e., trauma, neoplasia, ‘other sick’, vaccination, ‘other healthy’ or post-operative check-up). Specifically, the definition provided to participating veterinary surgeons to categorise the animal presentation as GI disease is that the main reason for the animal’s presentation are signs including but not limited to diarrhoea, vomiting, weight loss and poor appetite. A full description of the SAVSNET data collection protocol has been described by Sánchez-Vizcaíno *et al*.^9^. The data for this study were gathered on a consultation-by-consultation basis, and include the date the animal was seen, unique identifiers for practice, premise and animal, the animal description (including species, breed, sex and date of birth), the syndromic level classification and the full postcode of each veterinary premise and pet owner.

Data were only gathered if the owner had not opted out of study participation. The collection and use of these data were approved by the University of Liverpool’s Research Ethics Committee (RETH00964); as such all collection and use of these data were performed in accordance with the relevant guidelines and regulations.

#### Data management

Text-based data for species and breed were cleaned to deal with misspellings or the use of non-standard terms by mapping to standard terms. A full description of this cleaning procedure has been described elsewhere^16^. Many breeds were present in the data set, some represented by only a few individuals, limiting the scope for analysis by breed. Thus, for the purposes of this study, only the animal’s classification as purebred or crossbred was used.

To identify localised outbreaks we needed to geocode all postcodes. The text-based data for each owner’s full postcode were automatically cleaned by applying mapping rules of typical misspellings (e.g. letter ‘O’ instead of zero). Any remaining records containing erroneous postcodes were discarded from our outbreak prediction as they could not be geocoded. Similarly, if the age of the animal was recorded outside the range 0 to 25 years then the record was excluded. SAVSNET records with missing data were removed before the analysis. If an animal attended a veterinary premise on more than one occasion during the study period we included all attendances without adjustment, on the grounds that multiple visits occurring within a short time period (e.g. within a few days) would likely indicate a more serious illness episode.

#### Data summary

Of the 1,211,326 consultations collected between 1^st^ March 2014 and 29^th^ February 2016, 72.3% were for dogs and 27.7% for cats. In 80.7% of all consultations a valid age, breed-status (purebred or crossbred) and owner’s full postcode were recorded; this subset of data is used for model selection and the basis for simulations. Gastrointestinal disease accounted for 4.0% of all presentations, amongst which 91.5% were recorded between Monday and Friday. Amongst animals presenting for GI disease, there was not a notable gender bias; 48.5% of dog consultations and 50.6% of cat consultations with a recorded sex were female. Where the breed-status was identified, 84.9% of dog GI disease consultations and 17.2% of cat GI disease consultations were purebreds. In animals with a date of birth recorded within the range 0 to 25 years, 65.4% of dog GI disease consultations and 47.4% of cat GI disease consultations were under eight years. The age profile of dogs and cats presenting for GI disease at SAVSNET veterinary premises stratified by sex and breed-status is shown in Table 1. Data for the two species were analysed separately.

**Table 1:** Age profile of dogs and cats attending SAVSNET veterinary premises for a gastrointestinal disease consultation stratified by sex and breed-status.

Species	Sex	Breed-status	Number of animal consultations by age category		
			<1 year	1 < 8 years	>=8 years
Dog	Female	Crossbred	429	1089	957
Dog	Female	Purebred	2266	6411	4969
Dog	Male	Crossbred	448	1151	916
Dog	Male	Purebred	2777	6876	4874
Cat	Female	Crossbred	488	1242	2295
Cat	Female	Purebred	123	233	403
Cat	Male	Crossbred	514	1319	1989
Cat	Male	Purebred	142	354	403

The number of dog and cat consultations shown included only animals with a mapped breed-status, sex and date of birth within the range of 0 to 25 years recorded.

### Measure of deprivation

We used the pet owner’s home postcode to assign a measure of deprivation to each owner using the most recent English^17^, Scottish^18^ and Welsh^19^ Indices of Multiple Deprivation (IMD) produced by their respective governments. A detailed description of how each government has developed their own measure of deprivation can be found elsewhere^20–22^. The three country-specific IMD measures are not directly comparable. We therefore included *country* as a three-level factor and rescaled the ranks of each country’s set of IMD scores to the range 0 to 1. For example, if for England the maximum rank was 32,000 and a location had rank 100 then the owner IMD explanatory variable would be assigned a value of 100/32,000.

## Outbreak Detection Modelling

### Rationale

As noted earlier, we define an *outbreak* as an unexplained spatially and temporally localised increase in the fraction of GI consultations amongst all consultations. The term “unexplained” refers to the fact that, for reasons that are well understood, some areas or times of year will experience higher fractions of GI consultations than others because of spatial variation in the local population susceptibility or temporal variation in the region-wide susceptibility to GI. We adjust for these known effects using measured explanatory variables, as described below in the section on explanatory variable selection. We then equate “unexplained” to “stochastic” and include this in our model as a latent, spatially and temporally correlated process *S*_*i*,*t*_, where *i* denotes premise and *t* denotes time, in days. By definition, the expected value of each *S*_*i*,*t*_ is zero, and our goal is to determine where and when its actual value is materially greater than zero. Note that the natural pattern of GI consultations will always be subject to fluctuations in time and space that cannot be explained fully by measured variables. It follows that outbreak detection is not a statistical hypothesis-testing problem. Our approach acknowledges this by the fact that the actual value of *S*_*i*,*t*_ will never be exactly zero. Our formal solution is therefore to calculate, for each premise *i* and day *t*, the predictive probability *q* (i.e. the probability conditional on all available data up to and including day *t*) that *S*_*i*,*t*_ > *l*, where *l* is a user-specified threshold representing an effect large enough to be of practical concern. We then declare an outbreak affecting premise *i* if this probability exceeds *q*_0_, the required positive predictive value per premise, say *q*_0_ = 0.95 or 0.99. As with any prediction problem using observational data, it is not possible simultaneously to control both the positive and negative predictive probabilities.

### Prediction model

To accommodate the spatial and temporal correlations that would characterise an outbreak of GI disease, we use a spatio-temporal mixed effects regression model, and fit the model using Bayesian inference. We define our binary response variable *Y*_*j*,*it*_ to take the value 1 if the *j*^*th*^ consultation at the *i*^*th*^ premise on day *t* is a GI disease presentation and 0 otherwise. Conditionally on an unobserved, spatio-temporally structured random effect *S*_*i*,*t*_, the *Y*_*j*,*i*,*t*_ are distributed as mutually independent Bernoulli variables with probabilities *p*_*j*,*i*,*t*_ defined by

1 $${\varPhi }^{-1}({p}_{j,i,t})={d}_{j,i,t}^{T}\theta +{S}_{i,t}$$

where ${\varPhi }^{-1}(\cdot )$ is the quantile function of the standard Normal distribution. The vector *d*_*j*,*i*,*t*_ denotes the set of explanatory variables and *θ* their associated regression parameters. We discuss selection of explanatory variables, *d*_*j*,*i*,*t*_, below.

The spatio-temporally structured collection of random effects for all premises and days is written as

2 $$S={({S}_{(1)}^{T},\ldots ,{S}_{(\tau )}^{T})}^{T}$$

where ${S}_{(t)}={({S}_{1,t},\ldots ,{S}_{n,t})}^{T}$and we denote by $\tau$ and *n*, respectively, the total numbers of days and premises contained in the data-set. The complete vector *S* follows a multivariate Normal distribution with mean zero and covariance matrix that incorporates the spatio-temporal context of the data. Specifically, we assume that, conditionally on its past, *S*_(*t*)_ follows a multivariate Gaussian distribution with mean vector $\phi {S}_{(t-1)}$ and spatial covariance matrix$\,\Omega$, which we construct as follows. Firstly, we associate with premise *i* a polygon consisting of all points closer to premise *i* than to any other premise; the resulting polygons, *V*_*i*_ are called Voronoi polygons. Secondly, we define the neighbours of *i* to be the set *N*(*i*) of premises whose Voronoi polygons are contiguous with *V*_*i*_. Finally, we define distance-decay weights

3 $${w}_{ik}=\{\begin{array}{c}{[1+{({u}_{ik}/\delta )}^{2}]}^{-1}\,\,{\rm{if}}\,k\in N(i),\,\,\delta  > 0\,\\ 0\,{\rm{otherwise}}\end{array}$$

where *u*_*ik*_ is the distance between premises *i* and *k*, and *δ* is a scaling parameter with units of distance. We then specify the conditional distribution of each *S*_*i*,*t*_ given all other *S*_*k*,*t*_ to be Normal with mean *ρm*_*it*_ where

4 $${m}_{it}=\frac{{\sum }_{k\in N(i)}{w}_{ik}{S}_{k,t}}{{\sum }_{k\in N(i)}{w}_{ik}},\,{\rm{for}}\,{\rm{all}}\,k\ne i$$

and variance ${\sigma }^{2}/{\sum }_{k\in N(i)}{w}_{ik}$. Together, these modelling assumptions imply that the so-called full conditional distributions of the *S*_*i*,*t*_ that together determine the joint distribution of *S* are of the form

5 $${S}_{i,t}|{S}_{k,t},\,{S}_{k,t-1} \sim N(\rho {m}_{it}+\phi \rho {m}_{it-1},\,\frac{{\sigma }^{2}}{{\sum }_{k\in N(i)}{w}_{ik}}),\,{\rm{for}}\,{\rm{all}}\,k\ne i$$

Using these full conditional distributions, we can simulate from the Bayesian predictive distribution of the random effects *S*_*i*,*t*_ using an MCMC algorithm based on auxiliary variable techniques as described in Section 4.3 of Rue & Held^23^. Our system is intended to be run in near-real-time, but the MCMC computations eventually become prohibitive as the time-span of the data, $\tau$, grows. To counteract this, we run the MCMC algorithm on a moving nine-day window, which is long enough to capture the temporal correlation in our data; the magnitude of the within-premise autocorrelation of *S*_*i*,*t*_ for a time lag of eight days is typically around 0.09. Over a time-window of this size, the effects of any systematic time-trend or seasonal effect on the fraction of GI consultations are negligible, which removes the need to include these as explicit terms in the model; see also section below on selection of explanatory variables.

We adopt the following set of mutually independent priors for the model parameters:

*θ* ~ MVN (0, 10^3^*I*); log *σ*^2^ ~ N (−5, 9); *ρ* ~ Uniform (0,1); *φ* ~ Uniform (0,1); *δ* ~ Uniform {1, 2, …, 100}

These were chosen to be vague, in the sense that they have little influence on the predictive inferences for the random effects *S*_*i*,*t*_ that constitute the primary goal of the analysis. However, if inferences about the model parameters are required, samples from their Bayesian joint posterior distribution are produced automatically as a by-product of the MCMC algorithm.

### Outbreak detection

Let *e*_*i*,*t*_ denote the exceedence probability for premise *i* on day *t*, i.e. the probability that *S*_*i*,*t*_ > *l* conditional on all available data up to and including day *t*, where *l* is the user-specified threshold value. To calculate the *e*_*i*,*t*_, we generate *M* posterior samples ${S}_{i,t}^{(1)},\ldots ,{S}_{i,t}^{(M)}$ from the joint predictive distribution of the random effects *S*_*i*,*t*_ using an MCMC algorithm, and calculate

6 $${e}_{i,t}=\frac{1}{M}\mathop{\sum }\limits_{m=1}^{M}{\rm{I}}({S}_{i,t}^{(m)} > l)$$

where ${\rm{I}}({S}_{i,t}^{(m)} > l)$ takes the value 1 if ${S}_{i,t}^{(m)} > l$ and 0 otherwise. For this calculation to be accurate, we need the MCMC algorithm first to run for a sufficiently long time, called the burn-in period, to have reached convergence and then for a further *M* iterations to feed Eq. (6), where *M* is sufficiently large that the sampling error on the right-hand-side of (6) is negligible. We used a burn-in period of 5,000 iterations, followed by *M* = 50,000 iterations.

The spatio-temporal model was fitted using the R package ‘caramellar’^24^.

### Explanatory variable selection

Generalised Linear Models (GLMs) are unsuitable for outbreak detection modelling because the parameter estimates and standard errors assume that the observations are independent; hence, they do not take account of spatial and/or temporal correlation. Nevertheless, we can use a standard probit regression model to establish whether there is a prima-facie case for including each explanatory variable in our outbreak prediction model, Eq. (1), using the following rule. We retained an explanatory variable if its effect was nominally significant at the conventional 5% level. This inclusion rule is conservative in the sense that in the presence of spatial or temporal correlation the standard probit regression analysis is likely to over-state the significance of individual regression effects. For both species, this led us to discard the explanatory variables pet insurance, micro-chipping and neutering status and to retain the following:

- the three-level factor ‘COUNTRY’ for the pet owner’s home address (i.e. England, Scotland or Wales);
- the two-level factor ‘WEEKDAY’ with values 0 and 1 indicating if the consultation date is a weekend day (Saturday, Sunday or public holiday) or a working weekday (Monday to Friday), respectively -we considered using day of the week as a factor on 7 levels, but this did not improve the fit significantly using a likelihood ratio (deviance difference) test;
- the two-level factor ‘GENDER’ with values 0 and 1 corresponding to ‘female’ and ‘male’, respectively;
- the two-level factor ‘PUREBRED’ with values 0 and 1 corresponding to crossbred or purebred, respectively;
- the continuous variable ‘AGE’ denoting the animal’s age, in years and AGE^2^ = AGE × AGE, both included because the quadratic term improves the model fit;
- the continuous variable ‘IMD’, is the rescaled deprivation measure relating to the pet owner’s home address (as described above in our section on data sources).

As noted earlier, fitting the model to moving nine-day windows of data removes any long-term trend or seasonal effects. The resulting provisional GLM is

7 $$\begin{array}{ccc}{\varPhi }^{-1}(p) & = & {\alpha }_{COUNTRY}+{\beta }_{COUNTRY}\times {\rm{IMD}}\,+{\theta }_{1}\,\times WEEKDAY+{\theta }_{2}\\  &  & \times {\rm{GENDER}}+{\theta }_{3}\times {\rm{PUREBRED}}+{\theta }_{4}\times {\rm{AGE}}+{\theta }_{5}\,\times AG{E}^{2}\end{array}$$

where $p$ denotes the probability that a presentation of a dog or cat (depending on the species evaluated) to a SAVSNET veterinary premise is recorded as a GI disease consultation. The first two terms on the right-hand side of Eq. (7) capture the interaction between country and IMD, so as to account for the fact that the three countries use different IMD measures, whilst ${\theta }_{1},\,{\theta }_{2},\ldots ,{\theta }_{5}$ are regression parameters for the remaining explanatory variables in the model. The GLM outputs for dogs and cats can be found as Supplementary Tables S1 and S2, respectively.

All computation was carried out using R version 3.4.0^25^.

## Outbreak Simulations

Our model’s ability to identify an outbreak, i.e. its sensitivity, is influenced by factors including the outbreak’s duration, spatial extent and the number of infected animals presenting at premises in the locality. In each of our simulations, we construct an outbreak by adding varying numbers of aberrant GI disease to the actual (baseline) SAVSNET-recorded cases in a specified set of premises over a specified number of consecutive days.

### Simulation model

We use the actual SAVSNET total consultations for dogs during February 2016, together with their associated explanatory variables, to simulate a step increase in the proportion of GI disease cases affecting one or more premises from a given day ${t}_{0},\,$corresponding to 15 February 2016, by augmenting Eq. (1) with an extra term as follows

8 $${\varPhi }^{-1}({p}_{j,i,t})={d}_{j,i,t}^{T}\theta +{S}_{i,t}+\gamma {{\rm{I}}}_{i}(t\ge {t}_{0}),$$

where the indicator function I_*i*_ for premise *i* has value 1 for premise *i* and all days $t\ge {t}_{0}$ if premise *i* is affected by the outbreak, and has value 0 otherwise. By varying the value of *γ* we can control the probability of a GI case at an affected premise.

For each simulation, we proceed as follows:

1. use the actual SAVSNET consultations during February 2016 to fit the no-outbreak model using Eq. (1) and to generate simulated realisations of *S*_*i*,*t*_;
2. for $t\ge {t}_{0}$, use the actual explanatory variables and the simulated *S*_*i*,*t*_ to compute *p*_*j*,*i*,*t*_ using Eq. (8) with $\gamma  > 0$;
3. use the computed values of *p*_*j*,*i*,*t*_ to simulate case and control flags (1 or 0 respectively) and use these to reassign each actual SAVSNET data consultation as either a case or control.

See Supplementary Material for detailed R-code.

### Simulation scenarios

**Figure 1:**
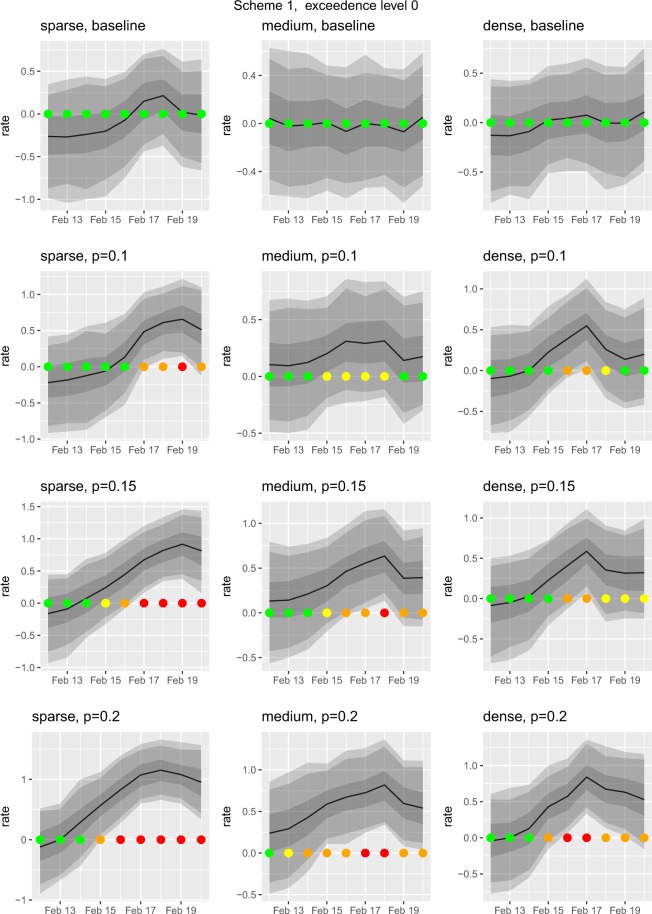
The results from our outbreak simulation study when using Scheme 1. In this scheme a single premise *i* at the centre of each region experiences an outbreak. Here we choose an exceedence level of *l* = 0 (see supplementary material for other levels). This figure shows the results of 9 simulations plus the baseline level. The top row of timeseries plots is the ‘baseline’, that is the actual SAVSNET data without any simulated outbreak i.e. *γ* = 0. The subsequent rows from top to bottom depict increasing severities of simulated outbreak labelled according the probability of a case at premise *i* e.g. *p* = 0 and so on. The columns, from left to right, relate to the density of the region; ‘sparse’, ‘medium’ and ‘dense’ respectively. For each simulation we plot the timeseries of the predicted distribution of *S*_*i*,*t*_ for premise *i*. In each time timeseries the solid black line is the predicted value of *S*_*i*,*t*_, shaded areas are pointwise 50%, 90% and 95% predictive intervals. As an aid to rapid interpretation, we use a traffic-light system: if the predictive probability, *q*, is above 0.99 (defined as ‘very high’) the light shows red, if above 0.9 (‘high’) orange, if above 0.8 (medium) yellow, otherwise (‘low’) green (no outbreak). The outbreak commences on 15^th^ February. The more intense the outbreak is the more the traffic light system tends towards red.

**Figure 2:**
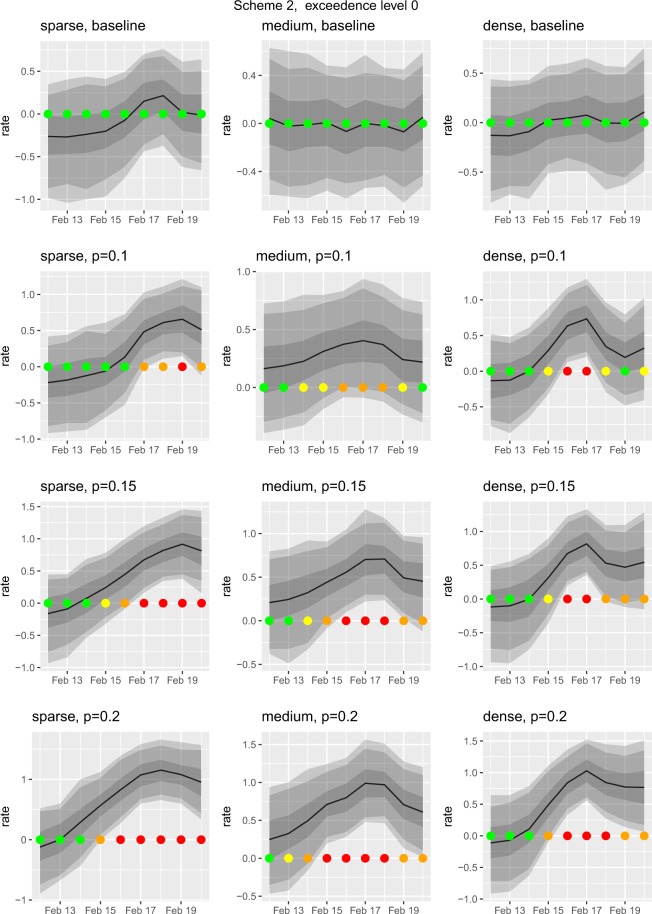
The results from our outbreak simulation study under Scheme 2. The overall layout and format of timeseries plots is the same as Fig. 1, for details see its caption. The simulated outbreak begins on 15^th^ February and the timeseries plots are for premise *i* at the centre of each region. Here we depict results using Scheme 2, that is premise *i *and its neighbours, within an 8 km radius, experience an outbreak. Again we choose an exceedence level *l* = 0 (see Supplementary Material for other levels).

We applied our simulation model to three *sets* of premises, which we selected based on their numbers of *neighbours*, defined to be other premises within an 8km radius, with the additional constraint that none of the sets of premises were within each other’s 8km radius. The selected sets of premises, which we designated as *dense*, *medium* and *sparse*, had 6, 3 and 0 neighbours, respectively. The SAVSNET data gave no indication that these selected premises are atypical or that they experienced a genuine outbreak during February 2016. See Figs 1 or 2, in each of which the top row, labelled ‘baseline’, is the actual SAVSNET data prior to simulating an outbreak. The premises at the centres of the three sets reported similar total numbers of consultations during February 2016 (349, 268 and 350 for dense, medium and sparse, respectively) and similar proportions of GI consultations (0.036, 0.055 and 0.042 for dense, medium and sparse, respectively). Using these three sets of premises, we simulated under 15 different scenarios as follows.

- Scheme 1: The outbreak only affects the central premise of each set. For each, we simulate outbreaks of different severities, in which the probability of a case is 0.1, 0.15 or 0.2. This gives a total of 9 scenarios.
- Scheme 2: The outbreak affects the central premise and all of its neighbouring premises. This leads to another 6 separate scenarios, as Schemes 1 and 2 are identical for the sparse set.

### Performance evaluation

We use each scenario to generate a simulated set of consultations for February 2016, to which we fit our model using Eq. (1). To assess the capability of our model to detect outbreaks we then use the predictive distribution *S*_*i*,*t*_ from which we compute summary statistics, including exceedence probabilities and times to detection. We set the positive predictive value of the system at *q*_0_ = 0.9. We set values of the reporting threshold at *l* = 0, 0.3 and 0.6. Note that *l* = 0 corresponds to an observed pattern exactly equal to expectation and is analogous to, although formally different from, using statistical rather than clinical significance in hypothesis testing. We do not recommend using *l* = 0 in practice, but use it here only as a benchmark to compare the system’s performance under different scenarios. In a genuine application, the threshold value *l* would be chosen to represent a clinically significant increase in reporting rate, and the positive predictive value *q*_0_ to balance sensitivity against specificity. Note, in this context, that because *Si,t* is measured on the probit scale, the increase in the fraction of GI cases corresponding to a fixed increase in *Si,t* necessarily depends on the baseline fraction. For example, if the expected fraction is 0.5, which corresponds to setting ${d}_{j,\,i,\,t}^{T}\theta =0$ and *S*_*i*,*t*_ = 0 in Eq. (1), then a log(2) threshold for *S*_*i*,*t*_ represents a fraction log(2) = 0.756 i.e. an increase of 0.256. In contrast, for a baseline fraction 0.1, a log(2) threshold now represents a fraction 0.278, i.e. an increase of 0.178.

### Simulation results

For each of the three regions (sparse, medium, dense) we ran our model a hundred times on the baseline data, where each run had a different random seed; we did not detect any false-positives with *l* = 0. Given the February 2016 baseline data, in Table 2 we report the credible intervals of the regression parameters estimated from the outbreak detection model’s MCMC samples.

**Table 2:** Regression parameters estimated by outbreak detection model given the baseline data during February 2016; our outbreak simulation results are based on this data.

quantile	weekday (weekend)	weekday (workday)	gender (male)	purebred	age	age^2^	IMD
0. 025	−1.8	−1.9	−0.010	−0.210	−0.071	8.1e-05	0.018
0.5	−1.6	−1.8	0.060	−0.120	−0.042	2.2e-03	0.160
0.975	−1.5	−1.7	0.140	−0.038	−0.012	4.2e-03	0.310

Note, the spatial overall domain of the outbreak simulations is the north west of England hence there is no country effect; see Eq. 7.

Our model detected a simulated outbreak in 14 out of the 15 outbreak scenarios when the reporting threshold was set at *l* = 0 (Table 3). The model detected an outbreak on the first day of its actual onset in six scenarios, one day after onset in a further seven scenarios and two days after onset in a further one scenario (Table 3). Alerting timeliness was inversely related to outbreak severity (Table 3).

**Table 3:** Timeliness of a spatio-temporal Bayesian mixed effects regression model at detecting a simulated outbreak in 15 different gastrointestinal disease outbreak scenarios, at a reporting threshold *l* = 0.

Spatial geometry	Extent	Severity (fraction of GI cases)	Timeliness (days to detection since start of outbreak)
Sparse	Confined to central premise	0.1	2
Sparse	Confined to central premise	0.15	1
Sparse	Confined to central premise	0.2	0
Medium	Confined to central premise	0.1	NA
Medium	Confined to central premise	0.15	1
Medium	Confined to central premise	0.2	0
Dense	Confined to central premise	0.1	1
Dense	Confined to central premise	0.15	1
Dense	Confined to central premise	0.2	0
Medium	Extending to neighbouring premises	0.1	1
Medium	Extending to neighbouring premises	0.15	0
Medium	Extending to neighbouring premises	0.2	0
Dense	Extending to neighbouring premises	0.1	1
Dense	Extending to neighbouring premises	0.15	1
Dense	Extending to neighbouring premises	0.2	0

In one scenario (NA: not applicable) timeliness could not be calculated because no outbreak was detected.

Figures 1 and 2 give a more detailed illustration of the performance of our outbreak detection methodology in response to a step change in the proportion of cases, for Schemes 1 and 2 respectively and with the threshold value *l* = 0. These figures also illustrate the use of a traffic-light system whereby, rather than fixing a single value for the positive predictive probability, *q*, we report a categorised value of the exceedence probabilities at each premise on each day to indicate the strength of the evidence for an outbreak.

**Figure 3:**
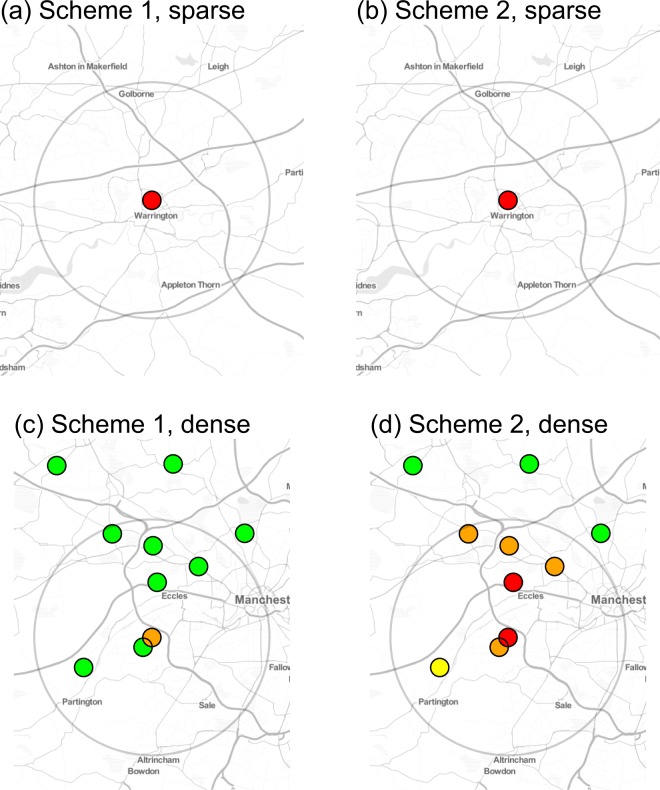
Maps of regions in which we simulated outbreaks where a premise is located at a coloured dot. These premises were selected for illustrative purposes, the actual SAVSNET data shows no indication that they are atypical or that they experienced a genuine outbreak during February 2016. As the base layer we use map tiles by Stamen Design, under CC BY 3.0: data by OpenStreetMap, under ODbL. The premise at the centre of each outbreak region is in the middle of the large light grey circle (8km radius). This figure shows the results of 4 simulations for 17^th^ February 2016 when we use an exceedence level of *l* = 0; n.b. the corresponding temporal results are given in Fig. 1. and 2. The top and bottom rows relate to the density of the region, ‘sparse’ and ‘dense’, respectively, and the left and right columns relate to simulation Schemes 1 and 2 respectively. The simulated probability of a case at the premise in the centre of each region is $p=0.15$. To aid interpretation, we use the traffic-light system described in Fig. 1 caption, as such each coloured circle on the map is derived from the predicted distribution of *S*_*i*,*t*_ at each corresponding premise. Panels (a,c) show when the central premise has neighbours who are not experiencing an outbreak it is less able to detect the outbreak, panel (c), when compared to a premise without neighbours, panel (a). If the neighbours also experience an outbreak the system is then better able to detect this outbreak at central premise, panel (d), compared with when the neighbours did not experience an outbreak, panel (c).

We focus on the sparse and dense sets of premises since the central premises of these two sets had almost identical numbers of consultations. Recall that under Scheme 1 the outbreak affects only the central premise of each set. Also, the prediction algorithm exploits the estimated spatial correlation amongst the fractions of GI cases at different premises. As a consequence, the system is better able to detect an outbreak at a single premise when this premise does not have close ‘outbreak-free’ neighbours whose fractions of GI cases are as expected. In effect, the model smooths its predictions over a range corresponding to its estimated correlation range; Fig. 3 shows an example of this phenomenon. This explains why, under Scheme 1 (Fig. 1), the system delivers a stronger detection signal for the sparse than for the dense set. Under Scheme 2 (Fig. 2), the results for the sparse and dense sets are more similar. Also, because the outbreak affects more premises in the medium, and dense sets, their results show generally stronger detection signals than in Scheme 1, as indicated by the increased number of traffic-lights tending towards red in Fig. 2 compared with Fig. 1.

Results of our model’s performance using the reporting thresholds *l* = 0.3 and *l* = 0.6 are available in the Supplementary Files; see Table S3 and Figs S1 and S3, and Table S4 and Figs S2 and S4, respectively. For example, given Scheme 1 (density sparse and *p* = 0.15) then: with *l* = 0 we detect an outbreak over the period 16 to 20 February (see Fig. 1); with *l* = 0.3 we also detect an outbreak, albeit less strongly, over the period 17 to 20 February (see Fig. S1 in Supplementary Material); with *l* = 0.6 we do not detect the outbreak (see Fig. S2). An increase in the reporting threshold value *l* necessarily reduces the probability that an outbreak will be declared and increases its time to detection (Tables S3 and S4, Figs S1–S4). This emphasises that the choice of *l* must be made in context and is unrelated to the inherent quality of the outbreak detection algorithm.

Setting the probability of a case to 0.1 and with *l* = 0, the model’s performance was compared with similar models in the sparse, medium and dense regions:

- *Model without covariates* ${\varPhi }^{-1}({p}_{j,i,t})={S}_{i,t}$. All the variation is accounted for by the latent term *S*_*i*,*t*_ so in a real-world application this model would be more prone to false-positives; in the context of Scheme 1 our simulations showed this model to be more sensitive. Comparing this model with the full model (Eq. 1) we find they are identical in terms of timeliness but the model without covariates shows more strength of the evidence for the outbreak in that the exceedence probabilities are higher overall.
- *Model without spatial correlation –* Scheme 1. In the presence of the outbreak only occurring at the central premise we found this model to be more sensitive at detecting outbreaks since the surrounding premises will not influence, and hence reduce, the inferred effects of the outbreak at the single central premise. Compared with the full model (with spatial correlation) we find this model to be identical in terms of timeliness for the sparse and dense regions, but the outbreak is now detected in the medium region with a one-day lag. Overall, the exceedence probabilities are higher in all regions.
- *Model without spatial correlation –* Scheme 2. With the outbreak spread over the neighbouring premises, this model was less sensitive as the neighbours did not influence, and therefore support, the detection of the outbreak. In particular we did not detect the outbreak in the medium and dense regions.

## Discussion

Syndromic surveillance systems offer the opportunity to enhance the public and animal health community’s ability to detect, and respond quickly to, disease outbreaks^5^. The last decade has seen a growth in the field of disease surveillance in companion animals, notably in the UK^9,26^ and in the USA^27,29^. However, to the best of our knowledge, this is the first surveillance system that conducts integrated spatio-temporal analysis of data from a national network of veterinary practices so as to enable real-time detection of spatially and temporally localised changes in reporting rate patterns across the network.

We have illustrated the applicability of our proposed surveillance system using gastrointestinal disease syndrome in dogs and cats as an example. The system is fed with electronic health records (EHRs) collected in real-time through SAVSNET from volunteer veterinary premises across the UK. We applied our system to 15 simulated GI disease outbreaks of varying spatial extent and severity, amongst which the system was able to detect 14 of the 15. Had these been real outbreaks, the proposed surveillance system would have triggered timely investigations, which ultimately would have aided control strategies. The system requires the user to specify a reporting threshold corresponding to an increase in case incidence (reporting rate) that would be considered large enough to be of practical importance. Given this reporting threshold, the system delivers the predictive probability, *q*, at each location (here, veterinary premise), that the threshold is currently exceeded. Declaring an outbreak when this probability is greater than a specified value *q*_0_ is equivalent to fixing the positive predictive value of the system (per location, per day) at *q*_0_. Alternatively, reporting the actual value of *q* gives an indication of the strength of evidence for an outbreak. Increasing the value of the reporting threshold, *l*, necessarily reduces the value of *q* and consequently increases the average time to detection of an outbreak at a fixed value of *q*_0._

A critical component of a syndromic surveillance system is the application of optimal disease aberration detection methods. Most of the methods used in veterinary and public health surveillance systems are concerned with detecting disease-outbreaks and health-related threats in time rather than in space^30–38^. However, disease incidences vary naturally in both space and time. Thus, for example, these techniques may be late at detecting outbreaks that start locally when the surveillance region is large^39^. In contrast, our proposed method has the advantage of being able to directly incorporate data for each individual animal’s consultation, including the date of the visit and the location of the pet’s owner. In temporal aberration detection algorithms, explanatory variables such as seasonality and day-of-the-week effects would generally be incorporated, but most of these methods cannot easily include individual-level explanatory variables.

Earlier spatio-temporal aberration detection methods have been introduced by Rogerson^40,41^. However, these approaches lack measures of uncertainty associated with the identified clusters and are unable to account for covariate information. Also, they are based on an assessment of global pattern change throughout the geographical area under study, as opposed to our method, which is used to detect the specific geographical location of an outbreak. Prospective space-time scan statistics have also been used in syndromic surveillance systems for the early detection of disease outbreaks^39,42^. The space-time permutation scan statistic uses only case numbers, with no need for population-at-risk data^39^ and, in contrast to Rogerson’s methods, does operate locally in both space and time. This method may therefore be suitable for setting up surveillance systems in the small animal sector where only case numbers are available. However, it does not acknowledge the uncertainty associated with any identified clusters, cannot easily incorporate continuous covariates, and can only detect outbreaks characterised by excess cases within a specified, regular shaped affected area, for example a circle or ellipse. Also, in our context the number of veterinary premises participating in SAVSNET can change over time due to the ongoing process of recruiting new premises and/or as a result of premises that could potentially stop being part of the project. This can lead to biased results if a space-time permutation model is used, as the method cannot distinguish an increase in cases due to a local population increase versus an increase in disease risk.

Our spatio-temporal model, in conjunction with a Bayesian inferential framework, takes account of all sources of uncertainty in both parameter estimation and prediction, and is able to accommodate spatial, temporal and individual-level covariate information. Other examples of Bayesian approaches include Markov models^43^, Bayesian information fusion networks^44^ and Bayesian hierarchical models^45–47^.

An earlier near-real-time syndromic surveillance system in small animals has been developed in the USA utilising EHRs from a similar network of primary care veterinary hospitals^29^. Briefly, in this approach the daily proportion of patients with a given clinical or laboratory finding was contrasted with an equivalent average proportion from a historical comparison period allowing construction of the proportionate diagnostic outcome ratio (PDOR)^29^. Our surveillance system builds upon a similar epidemiological metric by modelling the spatio-temporal reporting rate of GI disease in dogs and cats as a proportion of all presentations. The two approaches use different inferential methods: the US study uses confidence intervals for recognising aberrant health events, whilst our approach uses predictive probabilities of exceeding policy-relevant thresholds. A more important difference is that we use a bespoke model that incorporates spatio-temporal covariance structure, with the aim of detecting outbreaks that are spatially and temporally localised without imposing any artificial assumptions on the geometrical shape of an outbreak or the extent of spatial correlation in disease incidence.

Our inferential paradigm of predictive inference within a generalized linear mixed model could equally be applied in purely temporal surveillance settings where the aim is the timely detection of area-wide increases in reporting rate, but in that context we cannot claim the same level of novelty.

Another USA study explored the feasibility of using veterinary laboratory test orders as one of the data sources for syndromic surveillance in companion animals^28^. The inherent biases associated with the use of laboratory data in veterinary medicine have been described elsewhere^28,48–50^. However, the results derived from Shaffer *et al*.^28^ demonstrated the stability and timely availability of test order data for companion animals and the potential of using these data as a basis for outbreak detection. In addition to EHRs from veterinary practices, SAVSNET also receives routine downloads of diagnostic test results from commercial diagnostic laboratories throughout the UK^9^. Although laboratory test results are less timely than test orders, future research is warranted to explore whether the former data could be used to enhance the real-time syndromic surveillance system described here, which is based on real-time data from consultations in small animal premises.

Raising the reporting threshold, *l*, and/or the required positive predictive probability, *q*_*0*_, increases the specificity of the system at the cost of reducing its sensitivity, and conversely. In our analysis of the simulated outbreaks, we chose different reporting thresholds to illustrate the performance of our system. However, in any substantive application, the specified reporting threshold can and should be adjusted so as best to reflect end-users’ (i.e. veterinary surgeons in practice) preferred balance between sensitivity and specificity. A pragmatic choice would be to set the threshold to some proportion above the historic average at each premise.

End-users (hereafter “analysts”) of a real-time surveillance system will be responsible for receiving system outputs, interpreting them, and if necessary following up on alarms. Therefore, in addition to flexibility, another important attribute of a surveillance system should be that it reports outcomes in an easily interpretable manner. Our system generates outputs in the form of practice-specific time-series and maps that display the spatio-temporal evolution of GI disease risk over an area of interest in a user-friendly manner; see Fig. 3. Additionally, we have illustrated the use of a traffic-light device as a visual aid for analysts to quickly identify potential GI disease outbreaks on a given day at their own premises. The traffic-light device is based on predictive probabilities for exceedence of reporting thresholds that can be tailored to the analysts’ needs.

We intend to integrate our daily model-based predictions into the SAVSNET system so as to make them available to each participating premise through their SAVSNET web interface. This implementation will include the other two syndromes with outbreak potential that are currently recorded by SAVSNET (respiratory disease and pruritus). This syndromic surveillance system should be a step towards facilitating the prompt detection and control of health threats in companion animals throughout the UK. In addition, the identified temporal and geographical trends in specific syndromes can be a valuable contribution to the evidence-base when veterinarians are deciding how to treat individual animals in their practice.

One of the challenges of conducting epidemiological studies in the small animal sector is that information about the population-at-risk (in our study defined as the overall population of small animals across the UK or target population) is generally lacking. This makes it impossible to measure parameters typically used in human health surveillance systems, such as the average incidence in a day or period of days. Other methods must therefore be employed to approximate, for instance, an incidence rate ratio. Evidence suggests that in countries with developed pet industries, a high proportion of owned pet animals (pets who may approximate the target population) attend a veterinary surgeon^51,52^. Therefore, although no single data source can detect all outbreaks that may occur in companion animal populations, EHRs of the kind that are extensively collected from veterinary practices in many developed countries may be the best available source to include in surveillance activities for increasing our capabilities to detect those outbreaks that result from both endemic and potential emerging pathogens.

One limitation of this study is that the veterinary practices contributing data to our system were selected by convenience, based on their use of a compatible version of PMS, and recruited on the basis of their willingness to take part in the SAVSNET project. Hence, the data used in our system might not be representative of the source population (in our study defined as the overall veterinary-visiting population across the UK). For this reason, we aimed to develop a syndromic surveillance system to detect changes in the relative, rather than absolute, incidence of GI disease presentations in the small animal veterinary premises participating in SAVSNET. Nevertheless, the practices included in the current study were widely distributed around the UK and represented 8.5% of those practices that constituted the source population in 2009^51^. Thus, the number and geographical extent of SAVSNET-participating practices is such that changes in the relative risk of GI disease in this large network of premises can act as a proxy for changes in the level of GI disease in the wider source population.

A further limitation relates to missing data. Over the spatial domain and time-period of the simulation we found that 9% of consultations do not record location and 13% do not record breed. As a result, in total about 20% of the data are discarded due to incomplete data, our methodology assumes that these data are missing completely at random so that there is no inherent bias in the spatial distribution of the available data.

Another limitation is that each animal was classified only by its breed-status (purebred or crossbred). As such, we were unable to adjust for breed-specific phenotypes that could have an impact on the incidence of GI disease presentations. However, overall the breed distribution in our study population is consistent with previous studies. Labrador Retriever was the most common dog breed in our population as it is in earlier studies^8,51,53^. Also, nineteen out of the top twenty-six dog breeds in our study population were also in the top twenty breeds listed by The Kennel Club^53^. In future work we aim to identify additional means by which breeds can be effectively summarised according to both shared genotype and phenotype.

We are aware that the detection of a high relative risk for GI disease could trigger a false alarm if it is due to a localised decrease in the incidence of diagnosing other syndrome/s and routine veterinary interventions, leading to a higher than expected fraction of GI disease consultations. Conversely, a localised increase in the incidence of diagnosing other syndromes could conceal a genuine GI disease outbreak. If the goal is to detect anomalous patterns of absolute incidence rather than relative risk, then provided that data are available to calculate any changes in the population base of each premise our approach can be modified accordingly, for example by using a Poisson log-linear version of our spatio-temporal mixed model rather than the current binomial probit-linear version.

In order to understand and mitigate shared GI disease aetiologies between humans and animals it would be necessary to develop a ‘One Health’ surveillance system that integrates human and veterinary healthcare databases. In future work, we intend to adapt the approach described in this paper to human GI disease surveillance by re-calibrating the model against data relating to human GI disease presentations at general practitioner surgeries. A further extension of the approach would then be to a bivariate model for the joint surveillance of veterinary and human GI disease risk. A suitable starting point for this would be to replace the single Eq. (1) by a pair of equations,

9 $${\varPhi }^{-1}({p}_{j,i,t})={d}_{j,i,t}^{T}\theta +{S}_{i,t}$$

and

10 $${\varPhi }^{-1}(p{\text{'}}_{j,k,t})={e}_{j,k,t}^{T}\theta ^{\prime} +{S^{\prime} }_{k,t},$$

where Eqs. (9) and (10) describe the relative risk of GI at veterinary premise *i* and GP surgery *k*, respectively. A bivariate model would allow non-zero correlations between the *S*_*i*,*t*_ and ${S^{\prime} }_{k,t}$ corresponding to closely located pairs of veterinary premises and GP surgeries.

## Conclusions

We have demonstrated the feasibility of a real-time spatio-temporal syndromic surveillance system using as an example small animal veterinary premises in the UK. Our detection algorithm uses Bayesian predictive inference within a spatio-temporal model. The method demonstrated promising performance in detecting simulated outbreaks signals of varying spatial extent and severity at different reporting thresholds. The system is flexible: the reporting threshold of elevated risk and the positive predictive probability per premise and day may be set to whatever levels best meet the needs of a particular application; the system estimates the parameters of the model from historical data rather than imposing specific values for these, and can therefore be re-calibrated to detect outbreaks of any syndrome of interest. A traffic-light system based on exceedence probabilities offers a visual aid to rapid identification of potential outbreaks on a given day at each premise. We intend to implement the system on SAVSNET servers for the early detection of outbreaks in GI and in other syndromes that have outbreak potential and are routinely recorded in SAVSNET.

## Supplementary information

Supplementary Information

## Data Availability

The datasets generated and/or analysed during the current study are not publicly available due to issues of companion animal owner confidentiality, but are available on request from the SAVSNET Data Access and Publication Panel (savsnet@liverpool.ac.uk) for researchers who meet the criteria for access to confidential data. The R scripts used for pre-processing and analysing the data supporting this article can be found as Supplementary Material online. The R package ‘precara’ developed for pre-processing the data supporting this article is publicly available from the Zenodo repository (10.5281/zenodo.812822)^54^. The R package ‘caramellar’ developed to run the spatio-temporal model is publicly available from the GitHub repository (https://github.com/barryrowlingson/caramellar/tree/master)^24^.
