# Supplementary material for: A real-time spatio-temporal syndromic surveillance system with application to small companion animals
Source: Sci Rep. 2019 Nov 28;9:17738. doi: 10.1038/s41598-019-53352-6 (PMC6882870; doi:10.1038/s41598-019-53352-6)
Supplement: Supplementary file 1 — Supplementary Information [file 41598_2019_53352_MOESM1_ESM.pdf]

Supplementary information for

A real-time spatio-temporal syndromic surveillance system with application to small companion animals

Alison C. Hale[1*+], Fernando Sánchez-Vizcaíno[2*+], Barry Rowlingson[1], Alan D. Radford[3,4], Emanuele Giorgi[1], Sarah J. O'Brien[5,6], Peter J. Diggle[1]

[1] Centre for Health Informatics, Computing, and Statistics (CHICAS), Lancaster Medical School, Lancaster University, Lancaster LA1 4YW, UK

[2] Bristol Veterinary School, Langford Campus, University of Bristol, Bristol, BS40 5DU, UK

[3] NIHR Health Protection Research Unit in Emerging and Zoonotic Infections, University of Liverpool, UK

[4] Department of Infection Biology, Institute of Infection and Global Health, Leahurst Campus, University of Liverpool, Neston CH64 7TE, UK

[5] Department of Public Health and Policy, Institute of Psychology Health and Society, The Farr Institute@HeRC, University of Liverpool, Liverpool L69 3GL, UK

[6] NIHR Health Protection Research Unit in Gastrointestinal Infections, University of Liverpool, UK

* a.c.hale@lancaster.ac.uk

* f.s-vizcaino@bristol.ac.uk

[+] these authors contributed equally to this work

Contents of this file

Figures S1 to S4

Tables S1 to S4

Material A and B

Figure S1. Simulation results for Scheme 1 using an exceedance level of $l = 0.3$. The layout and formatting are identical to Figure 1.

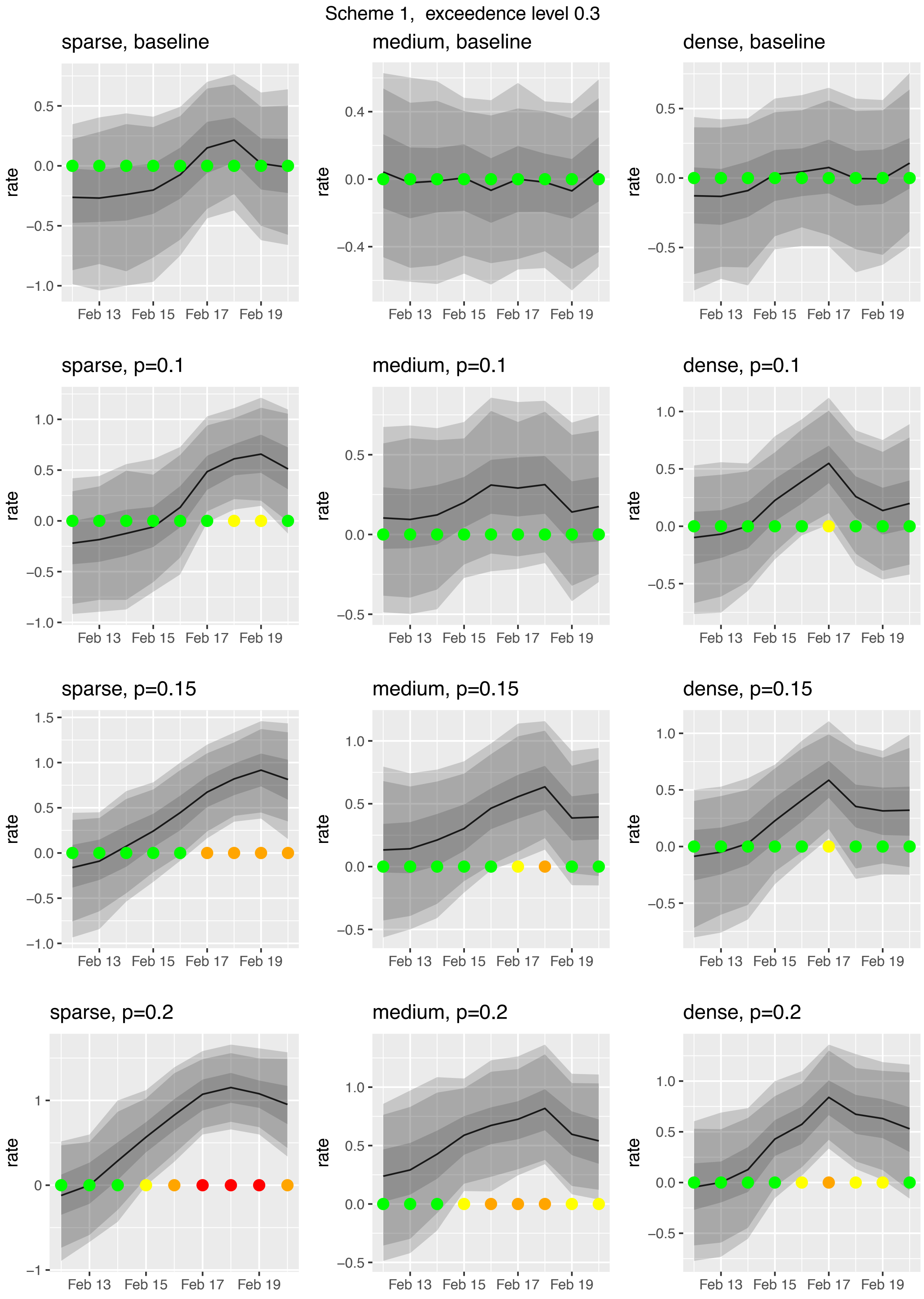

Figure S2. Simulation results for Scheme 1 using an exceedance level of $l = 0.6$. The layout and formatting are identical to Figure 1.

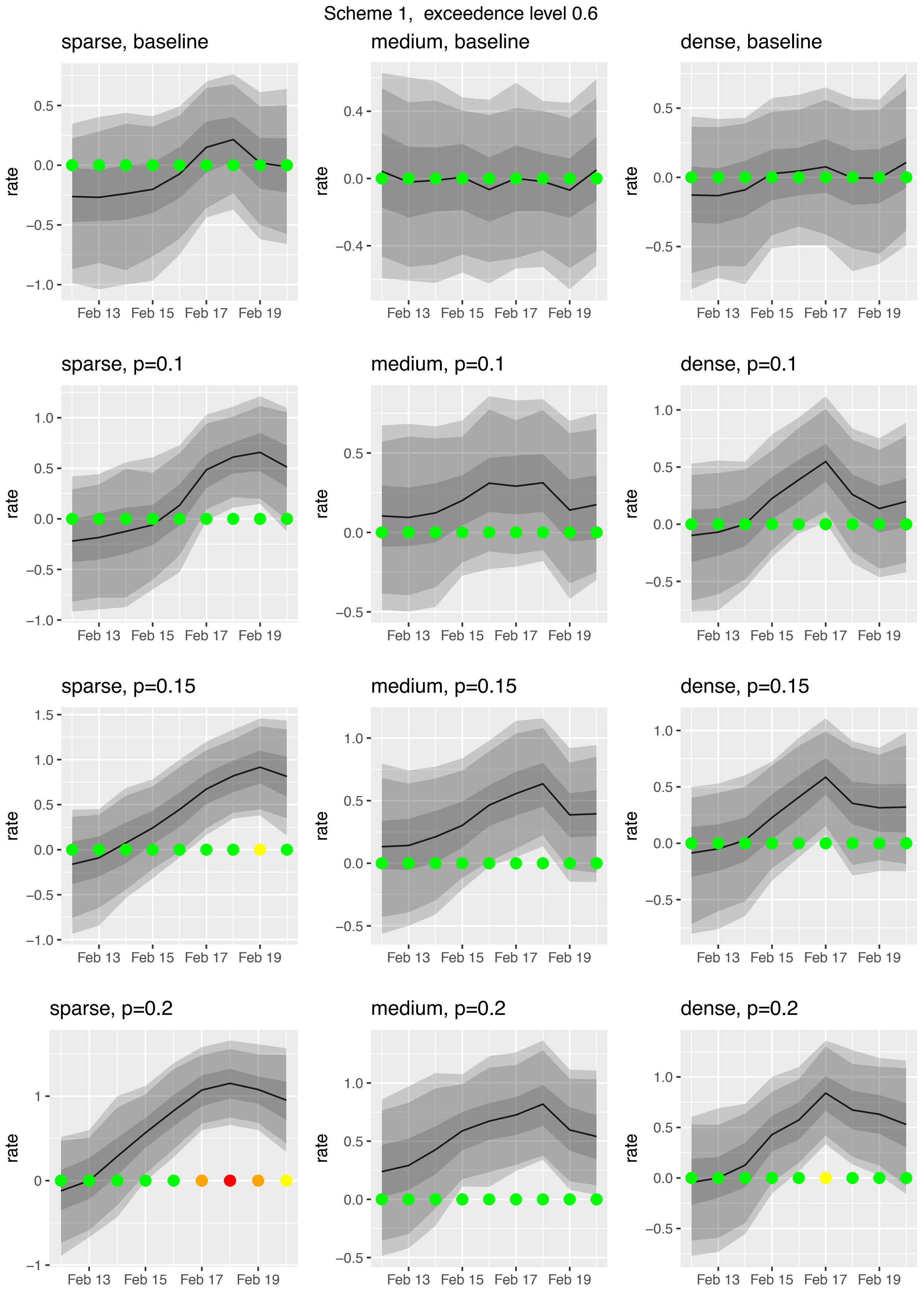

Figure S3. Simulation results for Scheme 2 using an exceedance level of $l = 0.3$. The layout and formatting are identical to Figure 1.

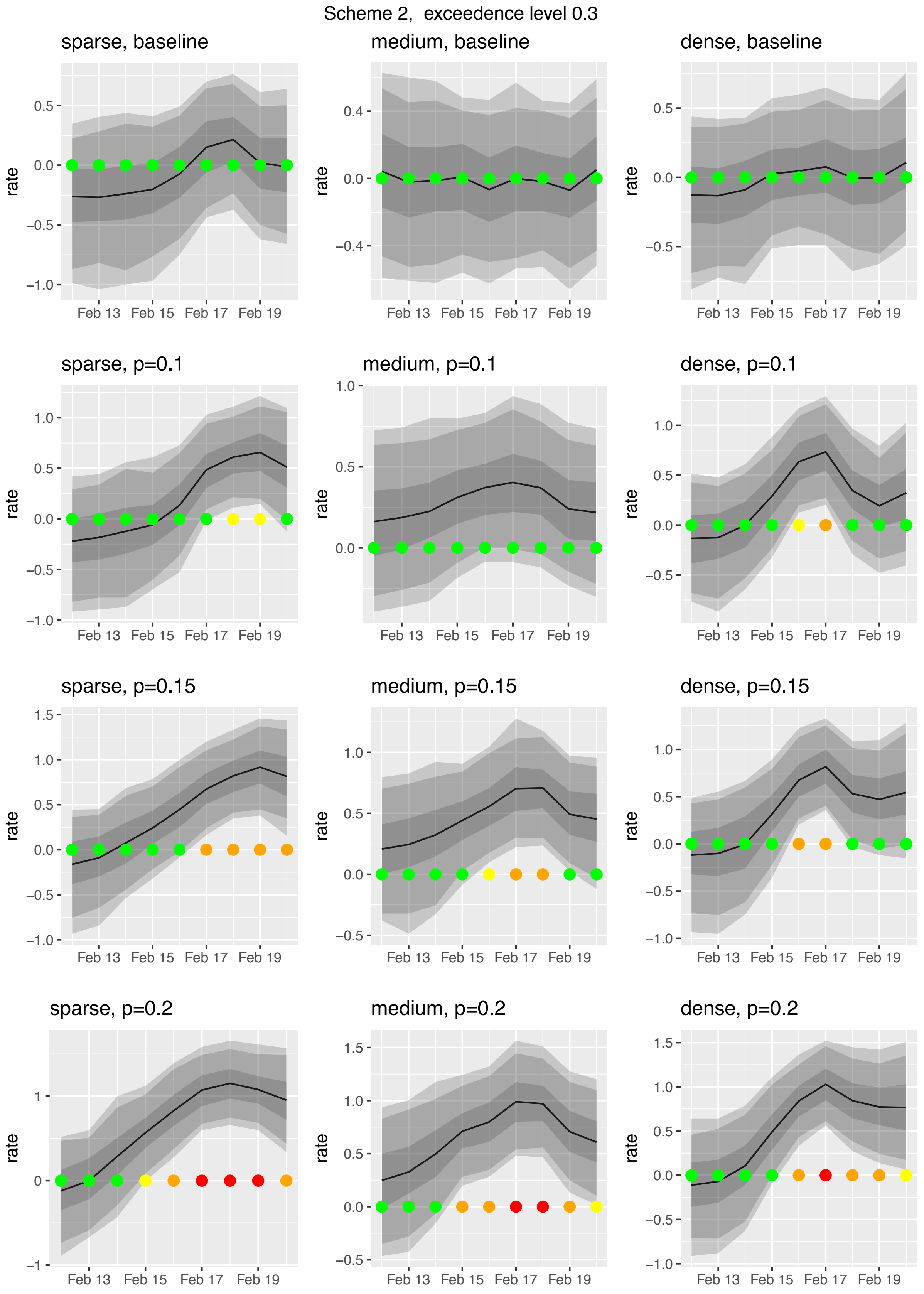

Figure S4. Simulation results for Scheme 2 using an exceedance level of $l = 0.6$. The layout and formatting are identical to Figure 1.

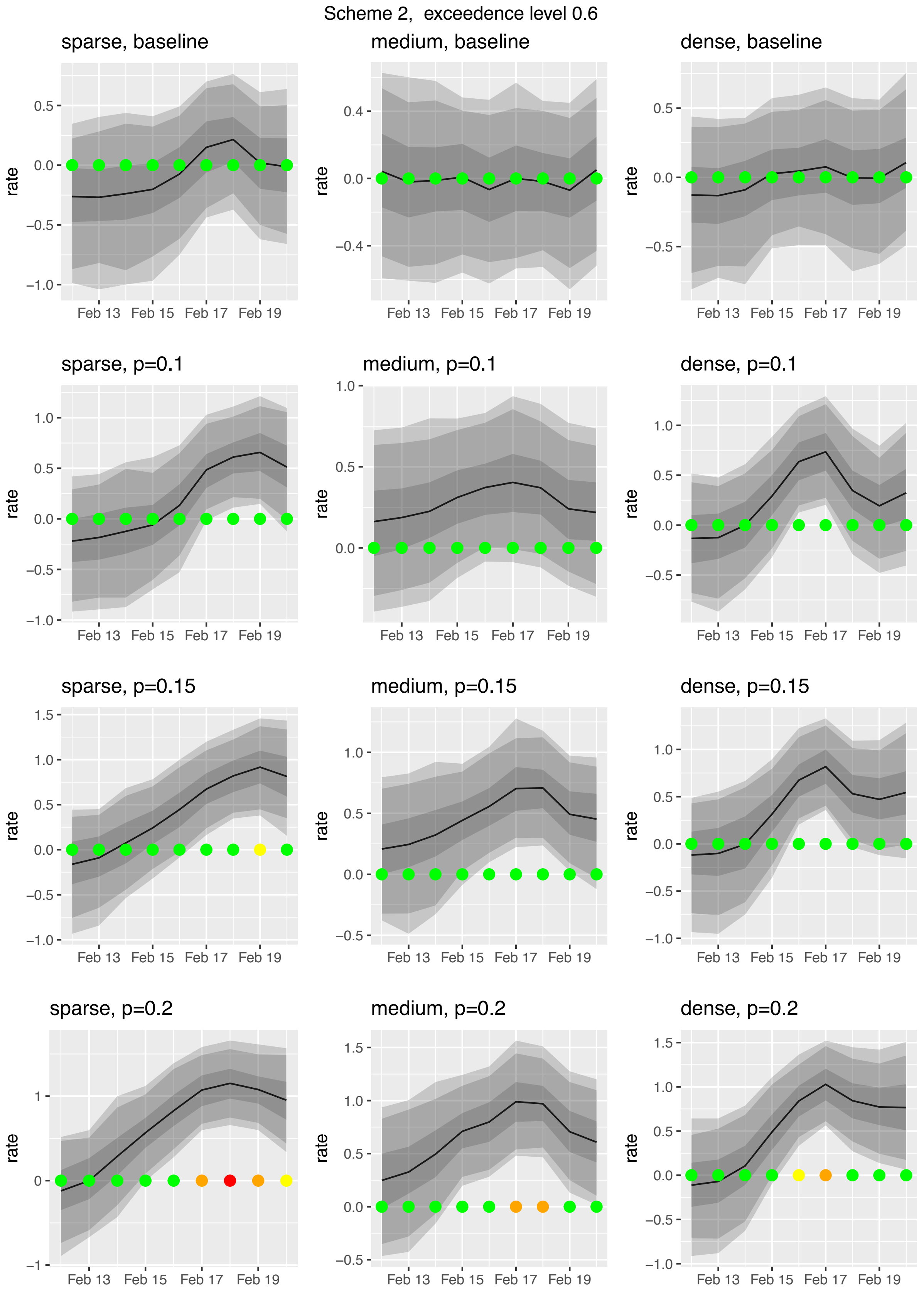

Table S1. Parameter estimates from a standard probit regression model for the probability of dogs presenting with gastrointestinal disease based on two years of SAVSNET data.

	Estimate	Std. Error	Z value	Pr (>|z|)
England	-1.6e+00	1.3e-02	-11.9e+01	< 2e-16
Scotland	-1.5e+00	2.9e-02	-5.3e+01	< 2e-16
Wales	-1.6e+00	2.5e-02	-6.4e+01	< 2e-16
weekday (1)	-2.1e-01	0.8e-02	-2.4e+01	< 2e-16
gender (male)	1.1e-02	0.5e-02	2.1e+00	3.9e-02
purebred (1)	3.1e-02	0.7e-02	4.2e+00	2.9e-05
age	-3.6e-02	2.0e-03	-1.8e+01	< 2e-16
age^2	2.5e-03	1.4e-04	1.8e+01	< 2e-16
England x IMD	1.6e-01	1.1e-02	1.5e+01	< 2e-16
Scotland x IMD	1.0e-01	4.1e-02	2.5e+00	1.2e-02
Wales x IMD	0.7e-01	3.8e-02	2.0e+00	4.6e-02

Table S2. Parameter estimates from a standard probit regression model for the probability of cats presenting with gastrointestinal disease based on two years of SAVSNET data.

	Estimate	Std. Error	Z value	Pr (>|z|)
England	-1.9e+00	2.1e-02	-9.5e+01	< 2e-16
Scotland	-1.8e+00	0.5e-01	-3.4e+01	< 2e-16
Wales	-1.9e+00	0.5e-01	-4.0e+01	< 2e-16
Weekday (1)	-0.7e-01	1.5e-02	-4.9e+00	8.9e-07
gender (male)	1.2e-02	0.9e-02	1.2e+00	2.0e-01
Purebred (1)	2.6e-01	1.3e-02	2.0e+01	< 2e-16
Age	1.4e-02	2.9e-03	4.6e+00	3.4e-06
age^2	-1.4e-04	1.6e-04	-0.9e+00	3.6e-01
England x IMD	0.7e-01	1.9e-02	3.9e+00	7.9e-05
Scotland x IMD	-1.7e-01	0.8e-01	-2.1e+00	3.5e-02
Wales x IMD	-0.8e-01	0.8e-01	-9.9e-01	3.2e-01

Table S3. Timeliness of a spatio-temporal mixed effects regression model at detecting a simulated outbreak in 15 different gastrointestinal disease outbreak simulations, at a reporting threshold $l = 0.3$.

Spatial geometry	Extent	Severity (fraction of GI cases)	Timeliness (days to detection since start of outbreak)
Sparse	Confined to premise *i*	0.1	NA[a]
Sparse	Confined to premise *i*	0.15	2
Sparse	Confined to premise *i*	0.2	1
Medium	Confined to premise *i*	0.1	NA
Medium	Confined to premise *i*	0.15	3
Medium	Confined to premise *i*	0.2	1
Dense	Confined to premise *i*	0.1	NA
Dense	Confined to premise *i*	0.15	NA
Dense	Confined to premise *i*	0.2	2
Medium	Confined to premise *i* and all neighbouring premises *j* within 8km radius	0.1	NA
Medium	Confined to premise *i* and all neighbouring premises *j* within 8km radius	0.15	2
Medium	Confined to premise *i* and all neighbouring premises *j* within 8km radius	0.2	0
Dense	Confined to premise *i* and all neighbouring premises *j* within 8km radius	0.1	2
Dense	Confined to premise *i* and all neighbouring premises *j* within 8km radius	0.15	1
Dense	Confined to premise *i* and all neighbouring premises *j* within 8km radius	0.2	1

[a] Not applicable: timeliness could not be calculated because the model did not detect any outbreak

Table S4. Timeliness of a spatio-temporal mixed effects regression model at detecting a simulated outbreak in 15 different gastrointestinal disease outbreak scenarios method, at a reporting threshold $l = 0.6$.

Spatial geometry	Extent	Severity (fraction of GI cases)	Timeliness (days to detection since start of outbreak)
Sparse	Confined to premise *i*	0.1	NA[a]
Sparse	Confined to premise *i*	0.15	NA
Sparse	Confined to premise *i*	0.2	2
Medium	Confined to premise *i*	0.1	NA
Medium	Confined to premise *i*	0.15	NA
Medium	Confined to premise *i*	0.2	NA
Dense	Confined to premise *i*	0.1	NA
Dense	Confined to premise *i*	0.15	NA
Dense	Confined to premise *i*	0.2	NA
Medium	Confined to premise *i* and all neighbouring premises *j* within 8km radius	0.1	NA
Medium	Confined to premise *i* and all neighbouring premises *j* within 8km radius	0.15	NA
Medium	Confined to premise *i* and all neighbouring premises *j* within 8km radius	0.2	2
Dense	Confined to premise *i* and all neighbouring premises *j* within 8km radius	0.1	NA
Dense	Confined to premise *i* and all neighbouring premises *j* within 8km radius	0.15	NA
Dense	Confined to premise *i* and all neighbouring premises *j* within 8km radius	0.2	2

[a] Not applicable: timeliness could not be calculated because the model did not detect any outbreak

Material A. R script used for pre-processing and selection of explanatory variables

```
################################################
### Exploratory analysis - general summary ###
################################################

rm(list=ls())
library(lubridate)

# SAVSNET data pre-cleaned by precara package returns variable robovetdata to the environment
load("./robovet_cleaned.RData")

DataFile  = "./SAVSNET_data.csv" # raw datafile (before any processing)
aa=read.csv(DataFile, head=TRUE)                                   # all raw data
GI=subset(aa, type_consult=="gastroenteric")                    # all GI
dogGI=subset(GI, species_cleaned=="dog")                        # all dog records with GI
catGI=subset(GI, species_cleaned=="cat")                      # all cat records with GI

cat("\nnumber of records (not including header) in raw datafile: ")
```

```
d = dim(aa)[1]
cat(d, "\n")

cat("\npercent records per species")
print(round(table(aa$species_cleaned)/sum(table(aa$species_cleaned))*100,1))

cat("\nnumber of records retained after cleaning ")
robovetdata_cleaned=robovetdata[complete.cases(robovetdata),]
cat(round(dim(robovetdata_cleaned)[1]/d*100,1), "\n")

cat("\npercent GI records\n")
print(data.frame(round(table(aa$type_consult)/sum(table(aa$type_consult))*100,1)))

cat("\npercent weekday\n")
IsWeekday=function(date) {
  daynum = lubridate::wday(as.Date(date, "%Y-%m-%d"))
  daynum[daynum==7]=1  # define sat or sun equal to equal 1
  daynum[daynum>1]=0   # define weekdays (mon-fri) to equal 0
  return(daynum)
}
is_weekend = IsWeekday(aa$consult_date)
print(round(table(is_weekend)/sum(table(is_weekend))*100,1))

cat("\nUsing only GI records: percentage of dog records by gender")
print(round(table(dogGI$gender)/sum(table(dogGI$gender))*100,1))

cat("\nUsing only GI records: percentage of cat records by gender")
print(round(table(catGI$gender)/sum(table(catGI$gender))*100,1))

cat("\nUsing only GI records: percentage of dog records by purebred")
print(round(table(dogGI$purebred)/sum(table(dogGI$purebred))*100,1))

cat("\nUsing only GI records: percentage of cat records by purebred")
print(round(table(catGI$purebred)/sum(table(catGI$purebred))*100,1))

cat("\nUsing only GI records: percentage of dogs records by age")
lowerage=0;
upperage=25;
dogAgesGI=dogGI;
dogAgesGI$age_years[dogAgesGI$age_years>=upperage] = NA
dogAgesGI$age_years[dogAgesGI$age_years<=lowerage] = NA
zz=ifelse(dogAgesGI$age_years<8, "under8","8+")
print(round(table(zz)/sum(table(zz))*100,1))
rm(zz)

cat("\nUsing only GI records: percentage of cats records by age")
catAgesGI=catGI;
catAgesGI$age_years[catAgesGI$age_years>=upperage] = NA
catAgesGI$age_years[catAgesGI$age_years<=lowerage] = NA
zz=ifelse(catAgesGI$age_years<8, "under8","8+")
print(round(table(zz)/sum(table(zz))*100,1))
rm(zz)

cat("\nstratified table dog")

ones=rep(1,dim(dogAgesGI)[1])
age1=1; age2=8;
dogdatasummary=data.frame(id=ones, age=dogAgesGI$age, purebred=dogAgesGI$purebred,
gender=dogAgesGI$gender)
dogdatasummary=dogdatasummary[complete.cases(dogdatasummary),];  # removes records with missing data
```

```
dogdatasummary$age=ifelse(dogdatasummary$age<age1,0,dogdatasummary$age)
dogdatasummary$age=ifelse(dogdatasummary$age>=age1 &
dogdatasummary$age<age2,1,dogdatasummary$age)
dogdatasummary$age=ifelse(dogdatasummary$age>=age2,2,dogdatasummary$age)
dogdatasummary$age=as.integer(dogdatasummary$age)

d3=dplyr::summarise(group_by(dogdatasummary, age, gender, purebred),
p=round(100*sum(as.numeric(id)/dim(dogdatasummary)[1]),2))
dd3=reshape::cast(d3, gender + purebred  ~ age, value = "p");
colnames(dd3)=c("gender","purebred","age<1","1>=age<8","age>=8")
dd3 = cbind.data.frame(animal=rep("dog",4), as.data.frame(dd3))
cat("total records:", sum(data.frame(dd3[,4:6])))
print(dd3);

d4=dplyr::summarise(group_by(dogdatasummary, age, gender, purebred), p=sum(as.numeric(id)))
dd4=reshape::cast(d4, gender + purebred  ~ age, value = "p");
colnames(dd4)=c("gender","purebred","age<1","1>=age<8","age>=8")
dd4 = cbind.data.frame(animal=rep("dog",4), as.data.frame(dd4))
print(dd4)
cat("total records:", sum(data.frame(dd4[,4:6])))


cat("\n\nstratified table cat")

ones=rep(1,dim(catAgesGI)[1])
age1=1; age2=8;
catdatasummary=data.frame(id=ones, age=catAgesGI$age, purebred=catAgesGI$purebred,
gender=catAgesGI$gender)
catdatasummary=catdatasummary[complete.cases(catdatasummary),];  # removes records with missing data

catdatasummary$age=ifelse(catdatasummary$age<age1,0,catdatasummary$age)
catdatasummary$age=ifelse(catdatasummary$age>=age1 & catdatasummary$age<age2,1,catdatasummary$age)
catdatasummary$age=ifelse(catdatasummary$age>=age2,2,catdatasummary$age)
catdatasummary$age=as.integer(catdatasummary$age)

d3=dplyr::summarise(group_by(catdatasummary, age, gender, purebred),
p=round(100*sum(as.numeric(id)/dim(catdatasummary)[1]),2))
dd3=reshape::cast(d3, gender + purebred  ~ age, value = "p");
colnames(dd3)=c("gender","purebred","age<1","1>=age<8","age>=8")
dd3 = cbind.data.frame(animal=rep("cat",4), as.data.frame(dd3))
cat("total records:", sum(data.frame(dd3[,4:6])))
print(dd3);

d4=dplyr::summarise(group_by(catdatasummary, age, gender, purebred), p=sum(as.numeric(id)))
dd4=reshape::cast(d4, gender + purebred  ~ age, value = "p");
colnames(dd4)=c("gender","purebred","age<1","1>=age<8","age>=8")
dd4 = cbind.data.frame(animal=rep("cat",4), as.data.frame(dd4))
print(dd4)
cat("total records:", sum(data.frame(dd4[,4:6])))


############################################################
### Exploratory analysis - GLM and outbreak detection ###
############################################################

rm(list=ls())
require(devtools)
require(calibrate)
require(xtable)
```

```
require(plyr)
require(plotrix)
require(dplyr)
library(gridExtra)
library(gapminder)
library(ggplot2)
require(ggmap)
require(rgdal)
require(spam)
require(reshape)

ukgrid = "+init=epsg:27700"  # Eastings and Northings (British National Grid)
latlong = "+init=epsg:4326"  # Latitude and Longitude (WGS84)

animals   = NA                              # animal of interest
case_name = "gastroenteric"                  # syndrome of interest
DataFile  = "./SAVSNET_data.csv"                  # raw datafile (before any processing)

startdate = "2014-03-01"                    # start date
enddate   = "2016-02-29"                     # end date (typically there should be 15 days of data)

homedir=getwd()

setwd("./SAVSNET")
load_all("precara")     # preprocessing package

LSOAyearE = 2001              # use LOSA data for England produced in this year (data file in precara in extdata)
LSOAyearW = 2011               # use LOSA data for Wales produced in this year   (data file in precara in extdata)


### run precara package to clean SAVSNET data
robovetdata = PreProcess(DataFile, case_name, LSOAyearE, LSOAyearW,
                   startdate=startdate, enddate=enddate,
                   animals=animals, progress=TRUE)

dogdata=subset(robovetdata, animal=="dog")
catdata=subset(robovetdata, animal=="cat")

names(dogdata) <- gsub("is_weekendOrHol", "workday_", names(dogdata))
names(catdata) <- gsub("is_weekendOrHol", "workday_", names(catdata))
levels(dogdata$workday_)=c("no","yes")
levels(catdata$workday_)=c("no","yes")

names(dogdata) <- gsub("gender", "gender_", names(dogdata))
names(catdata) <- gsub("gender", "gender_", names(catdata))

names(dogdata) <- gsub("purebred", "purebred_", names(dogdata))
names(catdata) <- gsub("purebred", "purebred_", names(catdata))
levels(dogdata$purebred_)=c("no","yes")
levels(catdata$purebred_)=c("no","yes")

names(dogdata) <- gsub("owner_country", "owner_country_", names(dogdata))
names(catdata) <- gsub("owner_country", "owner_country_", names(catdata))

expr       = substitute(is_case ~ -1 + owner_country_ + workday_ + gender_ + purebred_ + age + I(age^2) +
owner_country_:owner_IMD  )

dogdataclean=dogdata[complete.cases(dogdata),]
glm_dog=stats::glm(expr, data=dogdataclean, family=binomial(link="probit"))
```

```
glm_dog_tbl=print(xtable(glm_dog, caption="Fitted GLM Parameters for Dogs", label="glmdog", digits=1,
display=c("s","e","e","e","e")), type="latex", caption.placement=getOption("xtable.caption.placement", "top"),
table.placement=getOption("xtable.table.placement", NULL), latex.environments =
getOption("xtable.latex.environments", NULL))
glm_dog_tbl=gsub("I\\(age\\\\verb\\|\\^\\|2\\)", "age\\$\\^2\\$", glm_dog_tbl);
glm_dog_tbl=gsub("workday", "wday", glm_dog_tbl);
glm_dog_tbl=gsub("purebred", "pure", glm_dog_tbl);
glm_dog_tbl=gsub("owner\\\\_", "", glm_dog_tbl);
glm_dog_tbl=gsub("country\\\\_", "", glm_dog_tbl);
glm_dog_tbl=gsub("England", "Eng", glm_dog_tbl);
glm_dog_tbl=gsub("Wales", "Wal", glm_dog_tbl);
glm_dog_tbl=gsub("Scotland", "Scot", glm_dog_tbl);
print(summary(glm_dog))

catdataclean=catdata[complete.cases(catdata),]
glm_cat=stats::glm(expr, data=catdataclean, family=binomial(link = "probit"))
glm_cat_tbl=print(xtable(glm_cat, caption="Fitted GLM Parameters for Cats", label="glmcat", digits=1,
display=c("s","e","e","e","e")), type="latex", caption.placement=getOption("xtable.caption.placement", "top"),
table.placement=getOption("xtable.table.placement", NULL), latex.environments =
getOption("xtable.latex.environments", NULL))
glm_cat_tbl=gsub("I\\(age\\\\verb\\|\\^\\|2\\)", "age\\$\\^2\\$", glm_cat_tbl);
glm_cat_tbl=gsub("workday", "wday", glm_cat_tbl);
glm_cat_tbl=gsub("purebred", "pure", glm_cat_tbl);
glm_cat_tbl=gsub("owner\\\\_", "", glm_cat_tbl);
glm_cat_tbl=gsub("country\\\\_", "", glm_cat_tbl);
glm_cat_tbl=gsub("England", "Eng", glm_cat_tbl);
glm_cat_tbl=gsub("Wales", "Wal", glm_cat_tbl);
glm_cat_tbl=gsub("Scotland", "Scot", glm_cat_tbl);
print(summary(glm_cat))
```

Material B. R script for outbreak simulations for Schemes 1 and 2

```
###
### Schemes 1 and 2 outbreak simulations
###

rm(list=ls())
library(devtools)
library(calibrate)
library(xtable)
library(plyr)
library(plotrix)
library(dplyr)
library(gridExtra)
library(gapminder)
library(ggplot2)
library(ggmap)
library(rgdal)
library(spam)
library(reshape)
library(data.table)
library(sp)
library(readr)
library(ggsn)
```

```
###
### find and plot premsises
###

map0 = ggmap::get_stamenmap(c(bottom=50, top=56, left=-5, right=2), zoom=6, maptype="toner-lite")
ggmap(map0)

map1 = ggmap::get_stamenmap(c(bottom=53.176543, top=54.281390, left=-3.122119, right=-0.419482),
zoom=9, maptype="toner-lite")
ggmap(map1)

map2 = ggmap::get_stamenmap(c(bottom=53.3, top=54.2, left=-2.7, right=-1.6), zoom=10, maptype="toner-
lite")
ggmap(map2)

ukgrid = "+init=epsg:27700"
latlong = "+init=epsg:4326"

setwd("/home/haleac/Documents/Packages/SAVSNET")
load_all("precara")     # preprocessing package

PCFile <- read_csv("~/Documents/Packages/SAVSNET/precara/inst/extdata/CodePointOpenPostcodes.csv")
SAVSNET_rawData <- read_csv("~/Data/RawVetData/robovet_04-03-16/SAVSNET_data.csv")
pc = unique(SAVSNET_rawData$postcode_premise)

lookup = CleanPostcodes(pc,TRUE)
lookup = unique(lookup)

pcdata =
data.table(read.table("~/Documents/Packages/SAVSNET/precara/inst/extdata/CodePointOpenPostcodes.csv",
as.is=TRUE, sep=",", header=TRUE))   # load Code-Point Open postcode data
setkey(pcdata,"postcode")

pcxy=PCtoXY(pcdata,lookup)
pcxy=data.frame(postcode_clean=pcxy$postcode_clean, x=pcxy$x, y=pcxy$y)
pcxy=pcxy[complete.cases(pcxy),]
pcxy$postcode_clean=as.character(pcxy$postcode_clean)

coords = cbind(Easting=pcxy$x,Northing=pcxy$y)
sp = SpatialPointsDataFrame(coords, data=data.frame(pcxy$postcode_clean), proj4string=CRS(ukgrid))
sp_LL = spTransform(sp, CRS(latlong)) # plot(sp_LL)
colnames(sp_LL@coords)[colnames(sp_LL@coords) == "Easting"] <- "Longitude"
colnames(sp_LL@coords)[colnames(sp_LL@coords) == "Northing"] <- "Latitude"
pcxy=cbind(pcxy,data.frame("Longitude"=sp_LL@coords[,"Longitude"],
"Latitude"=sp_LL@coords[,"Latitude"]))


temp.pcxy = cbind.data.frame(long=pcxy$Longitude, lat=pcxy$Latitude)
#print(sp_LL@proj4string)
mapPoints <- ggmap(map0) + geom_point(aes(x=Longitude, y=Latitude), data = pcxy, alpha = .5) +
        ggsn::scalebar(temp.pcxy, dist=100, dd2km=TRUE, model='WGS84')
print(mapPoints)


###
### Define simulation region based on premises using latlong coords
###
```

```
pcxySmall = pcxy[pcxy$Longitude<(-0.419482) & pcxy$Longitude>(-3.122119) & pcxy$Latitude>53.176543
& pcxy$Latitude<54.281390,] #latlong coords
NorthingLimsY = c(min(pcxySmall$y), max(pcxySmall$y)) # region in Norhting-Eastings coords
EastingLimsX = c(min(pcxySmall$x), max(pcxySmall$x))
  #mapPoints <- ggmap(map) + geom_point(aes(x=Longitude, y=Latitude, size ="1"), data = pcxySmall, alpha =
.5); print(mapPoints)

rm(coords); rm(pcdata); rm(PCFile); rm(pcxySmall); rm(SAVSNET_rawData); rm(lookup); rm(mapPoints);
rm(pc); rm(pcxy);
rm(sp); rm(sp_LL); rm(temp.pcxy)



###
### Use most recent SAVSNET data for simulation
###

DataFile  = "~/Data/RawVetData/robovet_04-03-16/SAVSNET_data.csv"
case_name = "gastroenteric"     # syndrome of interest
LSOAyearE = 2001                # use LOSA data for England produced in this year (data file in precara in extdata)
LSOAyearW = 2011                # use LOSA data for Wales produced in this year   (data file in precara in extdata)
startdate = "2016-02-01"        # start date
enddate   = "2016-02-29"        # end date
animals   = "dog"               # animal of interest

robovetdata = PreProcess(DataFile, case_name, LSOAyearE, LSOAyearW, startdate=startdate,
enddate=enddate, animals=animals, progress=TRUE)
rrdata = robovetdata[robovetdata$premise_x<max(EastingLimsX) &
robovetdata$premise_x>min(EastingLimsX) & robovetdata$premise_y>min(NorthingLimsY) &
robovetdata$premise_y<max(NorthingLimsY),]
rrdata = rrdata[complete.cases(rrdata),]
rrdata = rrdata[rrdata$owner_country=="England",] # gets rid of any rogue points where owner doesn't live in
England (this only relates to 4 consultations in our simulation)

df = data.frame(id=as.character(rrdata$premise_id), x=rrdata$premise_x, y=rrdata$premise_y,
stringsAsFactors=FALSE ) # plotting df
df = df[!duplicated(df[,c('id')]),]
coords = cbind(Easting=df$x, Northing=df$y)
sp = SpatialPointsDataFrame(coords, data=data.frame(df$id), proj4string=CRS(ukgrid))
sp_LL = spTransform(sp, CRS(latlong)) # plot(sp_LL)
colnames(sp_LL@coords)[colnames(sp_LL@coords) == "Easting"] <- "Longitude"
colnames(sp_LL@coords)[colnames(sp_LL@coords) == "Northing"] <- "Latitude"
df=cbind(df,data.frame("Longitude"=sp_LL@coords[,"Longitude"], "Latitude"=sp_LL@coords[,"Latitude"]))

temp.pcxy = cbind.data.frame(long=df$Longitude, lat=df$Latitude)
mapPoints <- ggmap(map0) + geom_point(aes(x=Longitude, y=Latitude), data=df, alpha=.5, size=2,
color="#0000FF") +
  ggsn::scalebar(temp.pcxy, dist=10, dd2km=TRUE, model='WGS84')
print(mapPoints)

mapPoints <- ggmap(map1) + geom_point(aes(x=Longitude, y=Latitude), data=df, alpha=.5, size=3,
color="#FF0000") +
  ggsn::scalebar(temp.pcxy, dist=10, dd2km=TRUE, model='WGS84', location="topright")
print(mapPoints)

PremiseCoords = dd = df
dd=dd[dd$Longitude>attr(map2, "bb")$ll.lon & dd$Longitude<attr(map2, "bb")$ur.lon &
dd$Latitude>attr(map2, "bb")$ll.lat & dd$Latitude<attr(map2, "bb")$ur.lat,]  # coords taken from map2
variables
PremiseCoords.pcxy = temp.pcxy = cbind.data.frame(long=dd$Longitude, lat=dd$Latitude)
```

```
mapPoints <- ggmap(map2) + geom_point(aes(x=Longitude, y=Latitude), data=df, size=2, color="#0000FF") +
                    geom_text(aes(x=Longitude, y=Latitude, label=id), data=df, hjust=1.2, color="#0000FF",
size=3) +
                    ggsn::scalebar(temp.pcxy, dist=8, dd2km=TRUE, model='WGS84', location="topleft",
st.size=2)
print(mapPoints)

rm(DataFile); rm(case_name); rm(LSOAyearE); rm(LSOAyearW); rm(animals); rm(mapPoints); rm(coords);
rm(sp); rm(sp_LL); rm(df); rm(robovetdata); rm(map0); rm(map1); rm(map2); rm(temp.pcxy)



###
### run caramellar to get parameters (beta, sigma, rho, etc.)
###
setwd("~/Documents/Packages/SAVSNET/caramellar")
load_all("~/Documents/Packages/SAVSNET/caramellar")  # mcmc package
#expr       = is_case ~ -1 + owner_country + is_weekend + gender + purebred + age + I(age^2) +
owner_country:owner_IMD
expr       = is_case ~ -1 + is_weekend + gender + purebred + age + I(age^2) + owner_IMD     # note have
dropped country effect as isn't relavent for this simulation which is England only
mcPars     = mcmc_control(n.sim=55000,  burnin=5000, thin=100, hthin=500)

cat("\nLONG - all Feb: start carmc at"); print(Sys.time())
coords     = unique(rrdata[,c("premise_id","premise_x","premise_y")])
neighbours = voronoi_adjacency(coords, premise_id~premise_x+premise_y, scale=1000)
start_time = Sys.time()
output_L    = carmc(cases=rrdata, space_time=premise_id~consult_date, linear_model=expr,
neighbours=neighbours, noEXP=TRUE, mc_control=mcPars)
cat(" - runtime: ", Sys.time()-start_time, "hours - "); print(Sys.time())
output_file=paste("/media/haleac/SecondHD/SAVSNET/AdditionalSimulationData/mcmc_run_from_",min(rrd
ata$consult_date),"_to_",max(rrdata$consult_date),".RData",sep="")
save(output_L, file = output_file)



###
### Simulate GMRF - make simulated outbreak data
###
  set.seed(14)
  startOutbreak = "2016-02-15"   # start outbreak on Monday 8 Feb 2016
  outbreakPracticeDenseRegion  = "P216"
  outbreakPracticeMediumRegion = "P346"
  outbreakPracticeSparseRegion = "P311"

  cases = rrdata  # all Feb dataset
  space_time = premise_id~consult_date
  linear_model=expr

  ## get the location IDs and case dates
  spaces_times <- model.frame(space_time, data=cases)
  realDates <- spaces_times[,2]         # date objects
  spaces_times[,2] <- as.numeric(spaces_times[,2]) # convert Date to number
  #spatial_ids <- unique(spaces_times[,1])
  spatial_ids = output_L$space # correct order is set in carmc lines 124 onwards (AdjMat=... inverse.perm=...
spatial_ids=...). This order is then used in sim.full.cond.FAST when doing the cholesky
  realPracticeIds <- spaces_times[,1]   # practice ids
  listPracticeIds <- unique(as.character(spaces_times[,1]))

  times <- spaces_times[,2]
```

```
n.x <- length(spatial_ids)


## Construct all the W matrices
delta.set = seq(1,100,1) #  mcmc$delta$values
ind.delta.curr = round(mean(output_L$delta))   # parameter delta

W.list <- Wlist(delta.set, neighbours, spatial_ids)

time <- sort(unique(times))
n.t <- length(time)
nb_time <- make_nb_time(n.t)

ndata <- nrow(cases)

ID.space.time <- IDspacetime(spaces_times,spatial_ids)

mod.glm <- glm(formula=linear_model, family=binomial(link = "probit"),data=cases, x=TRUE)
D <- mod.glm$x
beta.curr <- as.matrix(colMeans(output_L$beta)) #  parameter beta
beta.names <- names(colMeans(output_L$beta))
covariates.effect <- D%*%beta.curr

rho.curr = mean(output_L$rho)   # parameter rho
W.curr <- W.list[[ind.delta.curr]]
Q.curr <- create.Q(rho.curr,W.curr)

phi.curr = mean(output_L$phi)  # parameter phi

sigma2.curr = mean(output_L$sigma2) # parameter sigma2

Q.t.curr <- create.Q.t(phi.curr, n.t, nb_time)

Q <- kronecker(Q.t.curr,Q.curr)/sigma2.curr

S.it <- base::solve(base::chol(Q),rnorm(n.t*n.x))

model.sim <- function(alpha, PracID) {
  outbreak <- rep(0,ndata)
  outbreak[realDates >= startOutbreak & realPracticeIds==PracID] <- 1    # indicator function 1 for given
practice beyond t0
  eta <- alpha*outbreak + as.numeric(covariates.effect) + S.it[ID.space.time]
  p <- pnorm(eta)

  #y <- rbinom(ndata, 1, p )            ############## this simulates across all practices and time periods not
just the one we are interested in
  y = array(0,ndata)
  for(aa in 1:ndata){
    if (outbreak[aa]>0){
      y[aa] = rbinom(1, 1, p[aa] )       # just on outbreak at given practice in give time period
    } else {
      y[aa] = rrdata$is_case[aa]         # leave remaining practices and time before outbreak untouched
    }
  }
  return( y )
}

# sim.results.y.... are the simulated cases for the outbreak simulation
# aa=0.01;
# alpha=qnorm(aa)-qnorm(0.05);
```

```
  # ...also note: alpha=uniroot(function(x) pnorm(qnorm(0.05)+x)-aa,lower=-5,upper=5)$root;  # equivalent to subtracting the qnorms

  realMean = 0.05  # set the mean at 0.05 so that the 2x, 3x, 4x, etc give prob if case as 0.1, 0.15, 0.2 etc.  This is a choice to reduce complexity in results and make comparing practices easier.
  #actualmean = mean(rrdata$is_case) # actual mean of gastro cases in dogs during Feb at given practice

  # simulated outbreak ***basekline*** number of cases
  alpha = 0  # baseline: here alpha is, and should be, zero
  set.seed(20); sim.results.y.dense.0x  = model.sim(alpha=alpha, PracID=outbreakPracticeDenseRegion )  # outbreakPracticeDenseRegion = "P388"
  set.seed(30); sim.results.y.medium.0x = model.sim(alpha=alpha, PracID=outbreakPracticeMediumRegion)  # outbreakPracticeMediumRegion = "P350"
  set.seed(40); sim.results.y.sparse.0x = model.sim(alpha=alpha, PracID=outbreakPracticeSparseRegion)  # outbreakPracticeSparseRegion = "P527"

  # set number of cases to be ***0.05*** for all practices ***
  set.seed(20); sim.results.y.dense.1x  = model.sim(alpha=alpha,      PracID=outbreakPracticeDenseRegion )  # outbreakPracticeDenseRegion = "P388"
  set.seed(30); sim.results.y.medium.1x = model.sim(alpha=alpha+0.15, PracID=outbreakPracticeMediumRegion)  # outbreakPracticeMediumRegion = "P350"
  set.seed(40); sim.results.y.sparse.1x = model.sim(alpha=alpha+0.17, PracID=outbreakPracticeSparseRegion) # outbreakPracticeSparseRegion = "P527"

        # set.seed(20); sim.results.y.dense.1xExtra1 etc. not needed as alpha=0 for dense practices

  set.seed(30); sim.results.y.medium.1xExtra1 = model.sim(alpha=alpha+0.15, PracID="P352")  # outbreakPracticeMediumRegion = "P350"
  set.seed(30); sim.results.y.medium.1xExtra2 = model.sim(alpha=alpha+0.15, PracID="P348")  # outbreakPracticeMediumRegion = "P350"
  set.seed(30); sim.results.y.medium.1xExtra3 = model.sim(alpha=alpha+0.15, PracID="P207")  # outbreakPracticeMediumRegion = "P350"
  sim.results.y.medium.1x = sim.results.y.medium.1x + sim.results.y.medium.1xExtra1 +
                            sim.results.y.medium.1xExtra2 +
                            sim.results.y.medium.1xExtra3
  sim.results.y.medium.1x[sim.results.y.medium.1x>1]=1


  # simulated outbreak ***double*** number of cases (0.1) ***
  alpha = qnorm(2*realMean)-qnorm(realMean);  # double cases on prob scale
  set.seed(20); sim.results.y.dense.2x  = model.sim(alpha=alpha, PracID=outbreakPracticeDenseRegion )  # outbreakPracticeDenseRegion = "P388"
  set.seed(30); sim.results.y.medium.2x = model.sim(alpha=alpha+0.28, PracID=outbreakPracticeMediumRegion)  # outbreakPracticeMediumRegion = "P350"
  set.seed(40); sim.results.y.sparse.2x = model.sim(alpha=alpha+0.17, PracID=outbreakPracticeSparseRegion) # outbreakPracticeSparseRegion = "P527"

  set.seed(20); sim.results.y.dense.2xExtra1  = model.sim(alpha=alpha, PracID="P389" )  # outbreakPracticeDenseRegion = "P388"
  set.seed(20); sim.results.y.dense.2xExtra2  = model.sim(alpha=alpha, PracID="P391" )  # outbreakPracticeDenseRegion = "P388"
  set.seed(20); sim.results.y.dense.2xExtra3  = model.sim(alpha=alpha, PracID="P388" )  # outbreakPracticeDenseRegion = "P388"
  set.seed(20); sim.results.y.dense.2xExtra4  = model.sim(alpha=alpha, PracID="P395" )  # outbreakPracticeDenseRegion = "P388"
  set.seed(20); sim.results.y.dense.2xExtra5  = model.sim(alpha=alpha, PracID="P393" )  # outbreakPracticeDenseRegion = "P388"
  set.seed(20); sim.results.y.dense.2xExtra6  = model.sim(alpha=alpha, PracID="P394" )  # outbreakPracticeDenseRegion = "P388"
  sim.results.y.dense.2x = sim.results.y.dense.2x + sim.results.y.dense.2xExtra1 +
```

```
                                    sim.results.y.dense.2xExtra2 +
                                    sim.results.y.dense.2xExtra3 +
                                    sim.results.y.dense.2xExtra4 +
                                    sim.results.y.dense.2xExtra5 +
                                    sim.results.y.dense.2xExtra6
  sim.results.y.dense.2x[sim.results.y.dense.2x>1]=1

  set.seed(30); sim.results.y.medium.2xExtra1 = model.sim(alpha=alpha+0.28, PracID="P352")  #
outbreakPracticeMediumRegion = "P350"
  set.seed(30); sim.results.y.medium.2xExtra2 = model.sim(alpha=alpha+0.28, PracID="P348")  #
outbreakPracticeMediumRegion = "P350"
  set.seed(30); sim.results.y.medium.2xExtra3 = model.sim(alpha=alpha+0.28, PracID="P207")  #
outbreakPracticeMediumRegion = "P350"
  sim.results.y.medium.2x = sim.results.y.medium.2x + sim.results.y.medium.2xExtra1 +
                                     sim.results.y.medium.2xExtra2 +
                                     sim.results.y.medium.2xExtra3
  sim.results.y.medium.2x[sim.results.y.medium.2x>1]=1

  # simulated outbreak ***tripple*** number of cases (0.15) ***
  alpha = qnorm(3*realMean)-qnorm(realMean);  # tripple cases on prob scale
  set.seed(20); sim.results.y.dense.3x  = model.sim(alpha=alpha, PracID=outbreakPracticeDenseRegion )  #
outbreakPracticeDenseRegion = "P388"
  set.seed(30); sim.results.y.medium.3x = model.sim(alpha=alpha+0.26,
PracID=outbreakPracticeMediumRegion)  # outbreakPracticeMediumRegion = "P350
  set.seed(40); sim.results.y.sparse.3x = model.sim(alpha=alpha+0.13, PracID=outbreakPracticeSparseRegion)
# outbreakPracticeSparseRegion = "P527"

  set.seed(20); sim.results.y.dense.3xExtra1  = model.sim(alpha=alpha, PracID="P389" )  #
outbreakPracticeDenseRegion = "P388"
  set.seed(20); sim.results.y.dense.3xExtra2  = model.sim(alpha=alpha, PracID="P391" )  #
outbreakPracticeDenseRegion = "P388"
  set.seed(20); sim.results.y.dense.3xExtra3  = model.sim(alpha=alpha, PracID="P388" )  #
outbreakPracticeDenseRegion = "P388"
  set.seed(20); sim.results.y.dense.3xExtra4  = model.sim(alpha=alpha, PracID="P395" )  #
outbreakPracticeDenseRegion = "P388"
  set.seed(20); sim.results.y.dense.3xExtra5  = model.sim(alpha=alpha, PracID="P393" )  #
outbreakPracticeDenseRegion = "P388"
  set.seed(20); sim.results.y.dense.3xExtra6  = model.sim(alpha=alpha, PracID="P394" )  #
outbreakPracticeDenseRegion = "P388"
  sim.results.y.dense.3x = sim.results.y.dense.3x + sim.results.y.dense.3xExtra1 +
                                    sim.results.y.dense.3xExtra2 +
                                    sim.results.y.dense.3xExtra3 +
                                    sim.results.y.dense.3xExtra4 +
                                    sim.results.y.dense.3xExtra5 +
                                    sim.results.y.dense.3xExtra6
  sim.results.y.dense.3x[sim.results.y.dense.3x>1]=1

  set.seed(30); sim.results.y.medium.3xExtra1 = model.sim(alpha=alpha+0.26, PracID="P352")  #
outbreakPracticeMediumRegion = "P350"
  set.seed(30); sim.results.y.medium.3xExtra2 = model.sim(alpha=alpha+0.26, PracID="P348")  #
outbreakPracticeMediumRegion = "P350"
  set.seed(30); sim.results.y.medium.3xExtra3 = model.sim(alpha=alpha+0.26, PracID="P207")  #
outbreakPracticeMediumRegion = "P350"
  sim.results.y.medium.3x = sim.results.y.medium.3x + sim.results.y.medium.3xExtra1 +
                                     sim.results.y.medium.3xExtra2 +
                                     sim.results.y.medium.3xExtra3
  sim.results.y.medium.3x[sim.results.y.medium.3x>1]=1

  # simulated outbreak ***quadruple*** number of cases (0.2) ***
  alpha = qnorm(4*realMean)-qnorm(realMean);   # quadruple cases on prob scale
```

```
  set.seed(20); sim.results.y.dense.4x  = model.sim(alpha=alpha+0.07, PracID=outbreakPracticeDenseRegion )
# outbreakPracticeDenseRegion = "P388"
  set.seed(30); sim.results.y.medium.4x = model.sim(alpha=alpha+0.43,
PracID=outbreakPracticeMediumRegion)
  set.seed(40); sim.results.y.sparse.4x = model.sim(alpha=alpha+0.18, PracID=outbreakPracticeSparseRegion)
# outbreakPracticeSparseRegion = "P527"

  set.seed(20); sim.results.y.dense.4xExtra1  = model.sim(alpha=alpha, PracID="P389" )  #
outbreakPracticeDenseRegion = "P388"
  set.seed(20); sim.results.y.dense.4xExtra2  = model.sim(alpha=alpha, PracID="P391" )  #
outbreakPracticeDenseRegion = "P388"
  set.seed(20); sim.results.y.dense.4xExtra3  = model.sim(alpha=alpha, PracID="P388" )  #
outbreakPracticeDenseRegion = "P388"
  set.seed(20); sim.results.y.dense.4xExtra4  = model.sim(alpha=alpha, PracID="P395" )  #
outbreakPracticeDenseRegion = "P388"
  set.seed(20); sim.results.y.dense.4xExtra5  = model.sim(alpha=alpha, PracID="P393" )  #
outbreakPracticeDenseRegion = "P388"
  set.seed(20); sim.results.y.dense.4xExtra6  = model.sim(alpha=alpha, PracID="P394" )  #
outbreakPracticeDenseRegion = "P388"
  sim.results.y.dense.4x = sim.results.y.dense.4x + sim.results.y.dense.4xExtra1 +
                                        sim.results.y.dense.4xExtra2 +
                                        sim.results.y.dense.4xExtra3 +
                                        sim.results.y.dense.4xExtra4 +
                                        sim.results.y.dense.4xExtra5 +
                                        sim.results.y.dense.4xExtra6
  sim.results.y.dense.4x[sim.results.y.dense.4x>1]=1

  set.seed(30); sim.results.y.medium.4xExtra1 = model.sim(alpha=alpha+0.43, PracID="P352")  #
outbreakPracticeMediumRegion = "P350"
  set.seed(30); sim.results.y.medium.4xExtra2 = model.sim(alpha=alpha+0.43, PracID="P348")  #
outbreakPracticeMediumRegion = "P350"
  set.seed(30); sim.results.y.medium.4xExtra3 = model.sim(alpha=alpha+0.43, PracID="P207")  #
outbreakPracticeMediumRegion = "P350"
  sim.results.y.medium.4x = sim.results.y.medium.4x + sim.results.y.medium.4xExtra1 +
                                         sim.results.y.medium.4xExtra2 +
                                         sim.results.y.medium.4xExtra3
  sim.results.y.medium.4x[sim.results.y.medium.4x>1]=1

  rm(cases); rm(space_time); rm(linear_model); rm(spaces_times); rm(realDates); rm(spatial_ids);
rm(realPracticeIds);
  rm(listPracticeIds); rm(times); rm(n.x); rm(delta.set); rm(ind.delta.curr); rm(W.list); rm(time); rm(n.t);
rm(nb_time); rm(ndata);
  rm(ID.space.time); rm(mod.glm); rm(D); rm(beta.curr); rm(covariates.effect); rm(rho.curr); rm(W.curr);
rm(Q.curr); rm(phi.curr);
  rm(sigma2.curr); rm(Q.t.curr); rm(Q); rm(S.it); rm(model.sim); rm(alpha);


  ###
  ### use simulated outbreak cases in caramellar
  ###

  drops = c("is_case")
  simdata = rrdata[ , !(names(rrdata) %in% drops)]

  outbreak_results = function(is_case, simdata, txt1, txt2, drops=NULL) {
    rrAll = cbind.data.frame(simdata, is_case=as.numeric(is_case))
    rrAll$syndrome[rrAll$is_case==0 & rrAll$syndrome=="gastroenteric"]="other_healthy"
    rrAll$syndrome[rrAll$is_case==1 & rrAll$syndrome!="gastroenteric"]="gastroenteric"
    # rrAll[!(rrdata$premise_id %in% drops), ]
```

```
  mcmc_period = 8                                    # number of days to including in each mcmc run
  leadlag = 1                                  # number of extra days before and after outbreak to run mcmc
  starting = as.Date(startOutbreak, "%Y-%m-%d") - as.difftime(leadlag+mcmc_period, unit="days")     # start
caramellar runs before outbreak starts
  ending   = as.Date(enddate, "%Y-%m-%d") - as.difftime(3, unit="days")               # end  caramellar runs
  dateseq = seq.Date(starting, ending, "days")          # date sequence

  for (i in 1:length(dateseq)){
    startday = dateseq[i]
    endday = startday + as.difftime(mcmc_period, unit="days")
    rr = subset(rrAll, (as.Date(consult_date, "%Y-%m-%d") >= startday & as.Date(consult_date, "%Y-%m-
%d") <= endday)) # get subset of data after startdate
    coords     = unique(rr[,c("premise_id","premise_x","premise_y")])
    neighbours = voronoi_adjacency(coords, premise_id~premise_x+premise_y, scale=1000)
    output     = carmc(cases=rr, space_time=premise_id~consult_date, linear_model=expr,
neighbours=neighbours, noEXP=TRUE, mc_control=mcPars)
    output_file =
paste("/media/haleac/SecondHD/SAVSNET/AdditionalSimulationData/mcmc_run_",txt1,"_",txt2,"_","from_",
min(rr$consult_date),"_to_",max(rr$consult_date),".RData",sep="")
    save(output, file = output_file)
  }

 }

  set.seed(103);  outbreak_results(sim.results.y.dense.0x,  simdata, "dense",  "alpha-zero") # simulated outbreak
data base line p(case)=0.05
  set.seed(104);  outbreak_results(sim.results.y.medium.0x, simdata, "medium", "alpha-zero")
  set.seed(105);  outbreak_results(sim.results.y.sparse.0x, simdata, "sparse", "alpha-zero")

  set.seed(103);  outbreak_results(sim.results.y.dense.1x,  simdata, "dense",  "alpha-one") # simulated outbreak
data base line p(case)=0.05
  set.seed(104);  outbreak_results(sim.results.y.medium.1x, simdata, "medium", "alpha-one")
  set.seed(105);  outbreak_results(sim.results.y.sparse.1x, simdata, "sparse", "alpha-one")

  set.seed(103);  outbreak_results(sim.results.y.dense.2x,  simdata, "dense",  "alpha-double") # simulated
outbreak data i.e. alpha>0
  set.seed(104);  outbreak_results(sim.results.y.medium.2x, simdata, "medium", "alpha-double")
  set.seed(105);  outbreak_results(sim.results.y.sparse.2x, simdata, "sparse", "alpha-double")

  set.seed(103);  outbreak_results(sim.results.y.dense.3x,  simdata, "dense",  "alpha-tripple")
  set.seed(104);  outbreak_results(sim.results.y.medium.3x, simdata, "medium", "alpha-tripple")
  set.seed(105);  outbreak_results(sim.results.y.sparse.3x, simdata, "sparse", "alpha-tripple")

  set.seed(103);  outbreak_results(sim.results.y.dense.4x,  simdata, "dense",  "alpha-quadruple")
  set.seed(104);  outbreak_results(sim.results.y.medium.4x, simdata, "medium", "alpha-quadruple")
  set.seed(105);  outbreak_results(sim.results.y.sparse.4x, simdata, "sparse", "alpha-quadruple")




###
### Plot time series
###

  TSclevels=c(.5,.90,.95)             # confidence levels (shaded grey regions on time series plots)
  TSelevels=c(0,0.3,0.6,0.8)            # e.g. quadruple, 0.8=qnorm(4*realMean)-qnorm(realMean), where real
mean is 0.05
  ev=1                         # which level to take out of TSelevels
  startday="2016-02-12"
  den = c("zero", "double", "tripple","quadruple")
```

```
  label = c("baseline", "p=0.1", "p=0.15", "p=0.2")
  for( j in 1:length(den) ){
    alpha=den[j]
    for ( i in 1:3 ){
      if (i==1) { practice=outbreakPracticeSparseRegion; density="sparse" }
      if (i==2) { practice=outbreakPracticeMediumRegion; density="medium" }
      if (i==3) { practice=outbreakPracticeDenseRegion;  density="dense"  }
      endday = as.Date(startday, "%Y-%m-%d") + as.difftime(mcmc_period, unit="days")
      load(paste("/media/haleac/SecondHD/SAVSNET/AdditionalSimulationData/mcmc_run_",density,"_alpha-
",alpha,"_from_",startday,"_to_",endday,".RData",sep=""))
      output$S = (output$S)
      ex = exceeds(output, output$startDate, TSclevels, TSelevels)
      plt = plot_exceeds(exceedences=ex, ev=ev, site_id=practice, traffic=TRUE,
title=paste(density,label[j],sep=", "), exp.s=FALSE)
      if (j==1 & i==1)  { p1=plt }
      if (j==1 & i==2)  { p2=plt }
      if (j==1 & i==3)  { p3=plt }
      if (j==2 & i==1)  { p4=plt }
      if (j==2 & i==2)  { p5=plt }
      if (j==2 & i==3)  { p6=plt }
      if (j==3 & i==1)  { p7=plt }
      if (j==3 & i==2)  { p8=plt }
      if (j==3 & i==3)  { p9=plt }
      if (j==4 & i==1) { p10=plt }
      if (j==4 & i==2) { p11=plt }
      if (j==4 & i==3) { p12=plt }
    }
  }
  pp = grid.arrange(p1, p2, p3, p4, p5, p6, p7, p8, p9,
                p10, p11, p12,
                ncol = 3, top=paste("Scheme 1, ","exceedence level",TSelevels[ev]))


###
### Make maps
###
  ev=1  # l=0
  alpha = den = "tripple"
  label = "p=0.15"
  endday = as.Date(startday, "%Y-%m-%d") + as.difftime(mcmc_period, unit="days")
  practice=outbreakPracticeSparseRegion; density="sparse"
  load(paste("/xxx/mcmc_run_",density,"_alpha-",alpha,"_from_",startday,"_to_",endday,".RData",sep=""))
  outputSparse=output
  load(paste("/xxx/mcmc_run_",density,"_alpha-",alpha,"_from_",startday,"_to_",endday,".RData",sep=""))
  outputSparseS2=output
  practice=outbreakPracticeDenseRegion;  density="dense"
  load(paste("/xxx/mcmc_run_",density,"_alpha-",alpha,"_from_",startday,"_to_",endday,".RData",sep=""))
  outputDense=output
  load(paste("/xxx/mcmc_run_",density,"_alpha-",alpha,"_from_",startday,"_to_",endday,".RData",sep=""))
  outputDenseS2=output

  ### make circle
  coordsSparse = PremiseCoords %>% filter(id=="P311")
  coordsDense  = PremiseCoords %>% filter(id=="P216")
  max_radius = 8 # 8km radius
  circleFun=function(center=c(0,0), r=1, n=100){
    pp = seq(0,2*pi, length.out=n)
    x = center[1] + r*cos(pp)
    y = center[2] + r*sin(pp)
    circle = data.frame(x=x, y=y)
```

```
   circle_SP  = SpatialPointsDataFrame(cbind(Easting  = circle$x, Northing = circle$y),
                              data=data.frame(Pid=paste("P",1:dim(circle)[1],sep="")),
                              proj4string = CRS(ukgrid))
   circle_SP_LL = spTransform(circle_SP, CRS(latlong))
   colnames(circle_SP_LL@coords)[colnames(circle_SP_LL@coords) == "Easting"]  = "Longitude"
   colnames(circle_SP_LL@coords)[colnames(circle_SP_LL@coords) == "Northing"] = "Latitude"
   circlepnts=cbind(circle,data.frame("Longitude" = circle_SP_LL@coords[,"Longitude"], "Latitude" =
circle_SP_LL@coords[,"Latitude"]))
   return(circlepnts)
  }
  circleSparse=circleFun(c(x=coordsSparse$x,y=coordsSparse$y), max_radius*1E3)
  circleDense =circleFun(c(x=coordsDense$x, y=coordsDense$y ), max_radius*1E3)

  # plot maps
  fLat = 0.002
  fLng = 0.06
  mapSparse = ggmap::get_stamenmap(c(bottom=(coordsSparse$Latitude)*(1-fLat),
top=(coordsSparse$Latitude)*(1+fLat),
                             left=(coordsSparse$Longitude)*(1+fLng) , right=(coordsSparse$Longitude)*(1-fLng)),
                             zoom=11, maptype="toner-lite")
  mapPointsSparse <- ggmap(mapSparse) + geom_point(aes(x=Longitude, y=Latitude), data=PremiseCoords,
size=2, color="#0000FF") +
    geom_text(aes(x=Longitude, y=Latitude, label=id), data=PremiseCoords, hjust=1.2, color="#0000FF",
size=3) +
    geom_path(data=circleSparse, aes(x = Longitude, y = Latitude), color="black", alpha = 0.5, size=1) +
    ggsn::scalebar(PremiseCoords, dist=8, dd2km=TRUE, model='WGS84', location="topleft", st.size=2)
  print(mapPointsSparse)

  mapDense = ggmap::get_stamenmap(c(bottom=(coordsDense$Latitude)*(1-fLat)+7*fLat,
top=(coordsDense$Latitude)*(1+fLat)+7*fLat,
                            left=(coordsDense$Longitude)*(1+fLng) , right=(coordsDense$Longitude)*(1-fLng)),
                            zoom=11, maptype="toner-lite")
  mapPointsDense <- ggmap(mapDense) + geom_point(aes(x=Longitude, y=Latitude), data=PremiseCoords,
size=2, color="#0000FF") +
    geom_text(aes(x=Longitude, y=Latitude, label=id), data=PremiseCoords, hjust=1.2, color="#0000FF",
size=3) +
    geom_path(data=circleDense, aes(x = Longitude, y = Latitude), color="black", alpha = 0.5, size=1) +
    ggsn::scalebar(PremiseCoords, dist=8, dd2km=TRUE, model='WGS84', location="topleft", st.size=2)
  print(mapPointsDense)

  ex    = exceeds(outputSparse, outputSparse$startDate, TSclevels, TSelevels)
  prems = join(data.frame(id=outputSparse$space), PremiseCoords,   by = NULL, type = "left", match = "all")
  p=ex$exceedence$p[[ev]][6,]
  pCat = cut(p, breaks=c(-1,.8,.9,.99,2),labels=c("Low","Medium","High","VHigh")) # exceedence categories
  pntdata1=cbind(prems,p=pCat)                              # data used to plot exceedences
  ppA=ggmap(mapSparse, extent = "panel", darken = c(.4,"white")) +
    geom_point(data = pntdata1, aes(x = Longitude, y = Latitude, col=p), alpha = 1, size=4) +
    scale_colour_manual(values=c("Low"="green","Medium"="yellow","High"="orange","VHigh"="red"),
                  limits=c("Low","Medium","High","VHigh"),
                  name="rate") + theme_void() + theme(legend.position="none") +
    geom_point(data = pntdata1, aes(x = Longitude, y = Latitude), alpha = 1, size=4, pch=21, col="black") +
    ggtitle('\n(a) Scheme 1, sparse') + theme(plot.title=element_text(hjust=0)) +
    geom_path(data=circleSparse, aes(x = Longitude, y = Latitude), color="black", alpha = 0.2)
  #print(ppA)

  ex    = exceeds(outputSparse, outputSparse$startDate, TSclevels, TSelevels)
  prems = join(data.frame(id=outputSparse$space), PremiseCoords,   by = NULL, type = "left", match = "all")
  p=ex$exceedence$p[[ev]][6,]
  pCat = cut(p, breaks=c(-1,.8,.9,.99,2),labels=c("Low","Medium","High","VHigh")) # exceedence categories
  pntdata1=cbind(prems,p=pCat)                              # data used to plot exceedences
```

```
ppB=ggmap(mapSparse, extent = "panel", darken = c(.4,"white")) +
  geom_point(data = pntdata1, aes(x = Longitude, y = Latitude, col=p), alpha = 1, size=4) +
  scale_colour_manual(values=c("Low"="green","Medium"="yellow","High"="orange","VHigh"="red"),
            limits=c("Low","Medium","High","VHigh"),
            name="rate") + theme_void() + theme(legend.position="none") +
  geom_point(data = pntdata1, aes(x = Longitude, y = Latitude), alpha = 1, size=4, pch=21, col="black") +
  ggtitle('\n(b) Scheme 2, sparse') + theme(plot.title=element_text(hjust=0)) +
  geom_path(data=circleSparse, aes(x = Longitude, y = Latitude), color="black", alpha = 0.2)
#print(ppB)

ex    = exceeds(outputDense, outputDense$startDate, TSclevels, TSelevels)
prems = join(data.frame(id=outputDense$space), PremiseCoords,   by = NULL, type = "left", match = "all")
p=ex$exceedence$p[[ev]][6,]
pCat = cut(p, breaks=c(-1,.8,.9,.99,2),labels=c("Low","Medium","High","VHigh")) # exceedence categories
pntdata1=cbind(prems,p=pCat)                          # data used to plot exceedences
ppC=ggmap(mapDense, extent = "panel", darken = c(.4,"white")) +
  geom_point(data = pntdata1, aes(x = Longitude, y = Latitude, col=p), alpha = 1, size=4) +
  scale_colour_manual(values=c("Low"="green","Medium"="yellow","High"="orange","VHigh"="red"),
            limits=c("Low","Medium","High","VHigh"),
            name="rate") + theme_void() + theme(legend.position="none") +
  geom_point(data = pntdata1, aes(x = Longitude, y = Latitude), alpha = 1, size=4, pch=21, col="black") +
  ggtitle('\n(c) Scheme 1, dense') + theme(plot.title=element_text(hjust=0)) +
  geom_path(data=circleDense, aes(x = Longitude, y = Latitude), color="black", alpha = 0.2)
#print(ppC)

ex    = exceeds(outputDenseS2, outputDenseS2$startDate, TSclevels, TSelevels)
prems = join(data.frame(id=outputDenseS2$space), PremiseCoords,   by = NULL, type = "left", match =
"all")
p=ex$exceedence$p[[ev]][6,]
pCat = cut(p, breaks=c(-1,.8,.9,.99,2),labels=c("Low","Medium","High","VHigh")) # exceedence categories
pntdata1=cbind(prems,p=pCat)                          # data used to plot exceedences
ppD=ggmap(mapDense, extent = "panel", darken = c(.4,"white")) +
  geom_point(data = pntdata1, aes(x = Longitude, y = Latitude, col=p), alpha = 1, size=4) +
  scale_colour_manual(values=c("Low"="green","Medium"="yellow","High"="orange","VHigh"="red"),
            limits=c("Low","Medium","High","VHigh"),
            name="rate") + theme_void() + theme(legend.position="none") +
  geom_point(data = pntdata1, aes(x = Longitude, y = Latitude), alpha = 1, size=4, pch=21, col="black") +
  ggtitle('\n(d) Scheme 2, dense') + theme(plot.title=element_text(hjust=0)) +
  geom_path(data=circleDense, aes(x = Longitude, y = Latitude), color="black", alpha = 0.2)
print(ppD)

pp=grid.arrange(ppA,ppB,ppC,ppD,nrow=2)
```
